# Resting state correlates of picture description informativeness in left vs. right hemisphere chronic stroke

**DOI:** 10.3389/fneur.2023.1288801

**Published:** 2023-12-07

**Authors:** Erin L. Meier, Shannon M. Sheppard, Rajani Sebastian, Shauna Berube, Emily B. Goldberg, Jennifer Shea, Colin M. Stein, Argye E. Hillis

**Affiliations:** ^1^Department of Neurology, Johns Hopkins University, Baltimore, MD, United States; ^2^Department of Physical Medicine and Rehabilitation, Johns Hopkins University, Baltimore, MD, United States; ^3^Department of Cognitive Science, Johns Hopkins University, Baltimore, MD, United States

**Keywords:** left hemisphere stroke, right hemisphere stroke, resting state, functional connectivity, discourse production, content units

## Abstract

**Introduction:**

Despite a growing emphasis on discourse processing in clinical neuroscience, relatively little is known about the neurobiology of discourse production impairments. Individuals with a history of left or right hemisphere stroke can exhibit difficulty with communicating meaningful discourse content, which implies both cerebral hemispheres play a role in this skill. However, the extent to which successful production of discourse content relies on network connections within domain-specific vs. domain-general networks in either hemisphere is unknown.

**Methods:**

In this study, 45 individuals with a history of either left or right hemisphere stroke completed resting state fMRI and the Cookie Theft picture description task.

**Results:**

Participants did not differ in the total number of content units or the percentage of interpretative content units they produced. Stroke survivors with left hemisphere damage produced significantly fewer content units per second than individuals with right hemisphere stroke. Intrinsic connectivity of the left language network was significantly weaker in the left compared to the right hemisphere stroke group for specific connections. Greater efficiency of communication of picture scene content was associated with stronger left but weaker right frontotemporal connectivity of the language network in patients with a history of left hemisphere (but not right hemisphere) stroke. No significant relationships were found between picture description measures and connectivity of the dorsal attention, default mode, or salience networks or with connections between language and other network regions.

**Discussion:**

These findings add to prior behavioral studies of picture description skills in stroke survivors and provide insight into the role of the language network vs. other intrinsic networks during discourse production.

## 1 Introduction

Discourse is the means through which people communicate their thoughts, feelings, and experiences during everyday conversation. Within formalist or structuralist frameworks, discourse is defined as the unit of language above the sentence level, whereas functionalist frameworks define discourse based on its capacity to meaningfully convey information within social contexts (1–4). Within the latter framework, a single word with significant meaning could be considered discourse (1), but more often, discourse samples contain multiple sentences that combine to form a coherent message. Discourse production likely relies on multiple cognitive systems, but the brain networks involved in different types of discourse genres (e.g., procedural, expository, narrative) remain underspecified.

Relevant to this topic is the fact that left and right hemisphere stroke survivors can exhibit discourse production impairments, albeit with different deficit profiles. Consistent with the notion that the left hemisphere (LH) is responsible for linguistic processing in most individuals, LH stroke survivors with aphasia often exhibit deficits in microstructural discourse properties, such as incorrect or omitted morphological and syntactic markers, reduced lexical diversity, and a decreased number of different word classes compared to normative samples [see reviews by Armstrong (1) and Linnik et al. (5)]. On the other hand, individuals with right hemisphere damage (RHD) most often demonstrate impaired macrostructural discourse skills such as poor discourse organization, tangentiality, and reduced local and global coherence, possibly due to cognitive-communication deficits in domains such as attention, executive functions, and pragmatics (6–8).

According to Armstrong (1), communication of meaningful information content (sometimes referred to as informativeness) falls in between micro- and macrostructural levels and may be deficient in individuals with a history of left or right hemisphere stroke. Discourse informativeness has been studied extensively via a variety of measures [e.g., content units, correct information units, lexical information units; see (5)] with mixed results. For example, some studies reported that people with aphasia (PWA) due to LH stroke produce significantly less meaningful discourse content, often with reduced efficiency, compared to neurologically healthy adults [e.g., (9–14)] while other studies reported no difference between PWA and controls or differences only for individuals with severe aphasia [e.g., (15–17)]. Similarly, lower informativeness scores in individuals with RHD compared to controls have been reported by some investigators [e.g., (9, 18–21)] but not others [e.g., (15, 16, 22)]. The type of information being conveyed may also matter, as there is some evidence that PWA demonstrate overall reduced lexical informativeness but not for main themes (10, 11), and content that requires inferential processing may be particularly susceptible in RHD (23, 24). Pertinent to this investigation is the fact that differences in discourse informativeness skills between individuals with LH damage (LHD) vs. RHD have not been established, partially due to the relative lack of studies [c.f. (15, 16, 19, 22)]. Given these inconsistent findings and the importance of informativeness in the success of discourse production, we focused on content unit (CU) measures derived from picture descriptions produced by our participants.

Currently, evidence regarding the neural substrates of discourse production impairments in left vs. right hemisphere stroke survivors is scant, and only a couple investigations (9, 16) have included measures that reflect the production of meaningful content. Using region-based lesion symptom mapping, Agis et al. (9) investigated which regions in the core LH language network and their RH homologs were implicated in reduced word-level content in acute left and right hemisphere stroke survivors, respectively. In the LH stroke group, they found that the total number of CUs produced when describing the Cookie Theft picture (25) was predicted by a model that included damage involving the left supramarginal gyrus (SMG), angular gyrus (AG), superior temporal gyrus (STG), middle temporal gyrus (MTG), and inferior temporal gyrus (ITG), as well as total lesion volume, but only damage to left ITG and lesion volume were significant independent predictors. While no single variable was significantly predictive in patients with acute RHD, a model that included infarct in the right inferior frontal gyrus, pars opercularis (IFGop), SMG, AG, STG, the superior longitudinal fasciculus, the sagittal striatum, and lesion volume was significant. Using whole-brain voxel-based morphometry, Schneider et al. (16) found that greater lexical informativeness during sequential scene descriptions was associated with greater gray matter density in the left primary sensory cortex and left insula—but not RH regions—across a sample of 10 patients with chronic LHD, 10 individuals with chronic RHD, and 10 neurologically healthy controls. In other lesion mapping studies that included LH stroke survivors with aphasia (but not individuals with RHD) (26–29), impaired production of meaningful discourse content was linked to damage to cortical regions spanning left frontal, temporal, and parietal lobes as well as several underlying LH white matter association tracts.

Collectively, lesion symptom mapping studies of informativeness deficits during discourse have primarily implicated LH structures which are traditionally associated with semantic or articulatory processes rather than RH regions or LH areas outside the canonical language network [cf. e.g., RH stroke findings in Agis et al. (9) and domain-general regions reported in Alyahya et al. (26)]. However, a major caveat to this conclusion is that most studies to date have been in individuals with post-stroke aphasia, and thus, the majority of analyses have been restricted to parts of the brain typically lesioned in PWA, i.e., language network regions within the left middle cerebral artery territory. Task-based functional imaging circumvents this issue but comes with added methodological limitations, such as stimulus-correlated motion artifacts in functional imaging time series data induced by overt speaking. As an alternative, resting state analysis allows for the delineation of intrinsic network markers that can then be correlated with participant performance on offline production tasks. Using such an approach, Duncan and Small (30) found that an increase in resting state network modularity from before to after an imitation-based therapy in 19 PWA was significantly associated with an increase in correct information units produced during retelling of the Cinderella story. In a follow-up investigation of dynamic functional connectivity, Duncan and Small (31) reported that treatment-related increases in correct information units were also significantly associated with increased dwell time in a state in which there was segregation of the default mode, dorsal attention, executive control, language, frontoparietal, sensorimotor, and visual networks. In other words, findings from these two studies indicate that treatment-induced improvements in discourse informativeness were associated with an improved balance between modularity and segregation of intrinsic domain-general and language-specific networks in PWA due to LH stroke.

The current study is motivated by the need to better understand the neurobiology of spoken discourse and the neural substrates that underlie impaired production of meaningful discourse content in individuals with LHD or RHD due to stroke. Our study had two aims. First, we evaluated differences between individuals with early chronic LH vs. RH stroke in three picture description measures derived from the Cookie Theft picture description task (25): (1) total number of CUs, primarily reflecting lexical-semantic skills; (2) the percentage of interpretative CUs, reflecting a combination of lexical-semantic and inferencing skills; and (3) the number of CUs produced per second, reflecting communication efficiency of meaningful scene content. Consistent with prior studies with overlapping measures [e.g., (9, 19, 24)], we hypothesized that individuals with LHD would produce fewer total CUs than individuals with RHD, but that several individuals within the RHD group would still demonstrate impaired CU production. Given the likely cause of discourse impairments following LH and RH stroke and the prior literature [e.g., (9, 12, 14, 23, 24)], we predicted that there would be no statically significant differences between groups in the percentage of interpretive CUs or communication efficiency of salient content.

Our second aim was to examine how picture description measures relate to resting state functional connectivity (rs-FC) of four intrinsic bilateral networks—the language network [LN; (32–36)], the core default mode network [DMN; (37–39)], the dorsal attention network [DAN; (40)], and the cingulo-opercular salience network [SN; (41)]—within LHD and RHD groups (Aim 2a) and between groups (Aim 2b). We interrogated these four networks to test the overarching hypothesis that the production of CUs within connected speech requires not only core linguistic processes mediated by the LN, but also domain-general cognitive skills processed within other intrinsic networks. More specifically, we hypothesized that all three CU measures would be positively associated with LN rs-FC in both groups, given that the production of CUs relies heavily on core stages of word retrieval (i.e., conceptual processing and semantic retrieval, lexical access, phonological retrieval and encoding, and articulation) and in some cases, morphosyntactic processes involved in the production of phrase-level utterances (42–47). We also predicted that all three measures would be positively associated with the within-network connectivity of other task-positive networks (i.e., DAN and SN) given that the DAN plays a role in directing attention to target stimuli to accomplish task goals (40, 48) and the SN is important for maintaining sustained attention to task (49–51). The DMN is considered a task-negative network which engages during rest but disengages during goal-directed activity (37, 39, 52). In other clinical populations (e.g., autism, dementia, schizophrenia), the integrity of the DMN is considered a biomarker for overall brain health (53–55). As such, we predicted that higher within-network connectivity of the DMN would likely also be related to better picture description ability in individuals with LHD or RHD [although cf. e.g., McCarthy et al. (56) and Weisssman et al. (57) for findings demonstrating worse behavioral outcomes coinciding with hyperconnectivity]. Given the importance of balanced network modularity and segregation in general and for discourse informativeness in prior studies in aphasia specifically (30, 31, 58), we expected that greater connectivity of between-network connections would be associated with worse CU scores. Finally, across networks, we predicted that relationships between picture description measures and left intra-hemispheric connectivity would be stronger in the RHD group than the LHD group whereas the opposite would be true of right intra-hemispheric connectivity.

## 2 Materials and methods

### 2.1 Participants

Data from 51 individuals (21 women; mean age of 57.4 ± 13.5 years) who completed a research MRI scan and behavioral testing as part of their participation in one of three ongoing studies at the Johns Hopkins University School of Medicine were considered for inclusion in the present study. Inclusion criteria were (1) a history of left or right cerebral hemisphere stroke at least 4 months prior to the time of MRI and testing, (2) pre-stroke proficiency in English, (3) normal or corrected-to-normal vision and hearing, and (4) no history of any neurological condition affecting cognition other than stroke. Individuals with multiple strokes were included if the affected tissue was primarily constrained to one cerebral hemisphere as indicated by a damage laterality index of <-0.8 for patients with RH stroke and > +0.8 for patients with LH stroke (see *2.4.1. Preprocessing* for additional details). Four potential participants were excluded due to laterality indices that fell outside of these limits. Two additional participants were excluded due to unusable MRI data.

The final sample comprised 45 stroke survivors (19 women; mean age of 57.9 ± 13.5 years), including 28 individuals with primarily LHD and 17 individuals with primarily RHD. Between-group comparisons indicated that the groups did not significantly differ in any demographic variable (Table 1). There were also no between-group differences in the time between the stroke onset that precipitated their study enrollment and their completion of MRI and testing procedures for the current investigation. See Table 1 for a summary of demographic and stroke data in each group and Supplementary Tables 1, 2 for these data for each participant.

**Table 1:** Comparisons of demographic and stroke characteristics between groups.

**Variable**	**LHD (*n =* 28)**	**RHD (*n =* 17)**	**Test statistic**	***P-v*alue**
Sex (*n* women)	15	4	3.64	0.066
Handedness (*n* right-handed)	27	14	0.180	0.144
Age (in years)	56.94 (14.19)	59.46 (12.44)	187.0	0.240
Education (in years)	14.04 (2.46)	14.35 (4.11)	216.5	0.759
Months Post-Onset	10.18 (3.560)	11.71 (6.78)	212.0	0.547
Total Lesion Volume (in mm^3^)	27,383.64 (50,315.53)	31,455.06 (53,037.61)	244.0	0.899
Laterality Index	0.98 (0.04)	−0.98 (0.06)	182.5	0.108
MCA Damage (*n* of sample)	17	13	0.483	0.341
Cortical Damage (*n* of sample)	20	12	1.041	1.000

Sex, handedness, and counts of participants with middle cerebral artery (MCA) and cortical involvement were compared using Fisher's exact tests; odds ratios are reported as the test statistic. The other variables were compared using Wilcoxon rank sum tests. For continuous variables, means and standard deviations (in parentheses) are reported. Statistical comparison of stroke laterality was performed used the absolute values of the laterality indices. The number of participants whose stroke occurred within the MCA territory and incurred at least some cortical damage is reported. LHD, left hemisphere damage; RHD, right hemisphere damage.

Study procedures were approved by the Institutional Review Board at Johns Hopkins University School of Medicine. All participants provided their written informed consent.

### 2.2 Language sample procedures

All participants completed the Cookie Theft picture description task from the Boston Diagnostic Aphasia Examination (25). Participants were instructed to provide a complete description of the depicted scene in their own words. All language samples were audio recorded and subsequently transcribed and scored by a research team member and speech-language pathologist (S.B.) with experience in language sample analysis. A second speech-language pathologist and research team member (E.G.) blinded to the participant ID, group assignment, and original scores scored a randomly selected subset of five samples from each group.

Completed transcripts included all utterances produced by the participant during the recording, including hesitations (e.g., “um”, “uh”), part-word repetitions (e.g., “b-boy”), whole word repetitions (e.g., “the boy boy”), and tangents unrelated to the picture. All utterances were considered in the sample scoring except for queries about the instructions or statements indicating the participant was done (e.g., “That's all I have to say”). The duration of each sample (in seconds), the total number of words, and the total number of syllables were calculated based on included utterances. Non-words (e.g., “kiplen”) were included in the syllable count but not the word count.

Our main measures of interest reflected communication of relevant scene content. To capture lexical-semantic abilities, the raters scored each sample for the total number of CUs per Yorkston and Beukelman (17). Each CU was counted only once even if the word was repeated or variations of the target word were used (e.g., *mother, mom, momma*). The percentage of CUs produced per second (CUs/second) was calculated to reflect communication efficiency of salient content. To measure inferencing abilities, the raters first counted the number of CUs that were included in Myers (24) list of interpretive concepts and then calculated the percentage of interpretative concepts by dividing the number of interpretive concepts by the total number of CUs and multiplying that value by 100. Although it was not a measure of primary interest, we also calculated the ratio of CUs in the left vs. right portion of the scene to capture potential hemispatial left neglect in the RHD group. Left to right CU ratios did not significantly differ between individuals with LHD vs. RHD (*W* = 221.5, *p* = 0.708).

To address Aim 1, we used Wilcoxon rank sum tests to compare the total number of CUs, percentage of interpretative CUs, and CUs/second between the LHD and RHD groups, correcting for multiple comparisons at a false discovery rate (FDR) of *q* < 0.05 for statistical significance. We also determined the number of participants within each group with impaired performance on each measure. Scores that fell more than 1.5 standard deviations below the control means reported in Yorkston and Beukelman (17) and Myers (24) were considered to reflect impairment.

Prior to completing Aim 1 analyses, we evaluated inter-rater reliability for the three CU measures of interest via intraclass correlations (ICC) for continuous data using the *irr* package (59) in R Studio (60). Inter-rater reliability was excellent for the total number of CUs [ICC = 0.960, *F*_(9, 10)_ = 48.40, *p* < 0.001, 95% CI 0.855 to 0.990] and CUs/second [ICC = 0.989, *F*_(9, 10)_ = 175.00, *p* < 0.001, 95% CI 0.958 to 0.997]. There was moderate reliability between raters for the percentage of interpretive CUs [ICC = 0.543, *F*_(9, 10)_ = 3.38, *p* < 0.036, 95% CI −0.056 to 0.861].

### 2.3 MR data acquisition

Participants were scanned at the F.M. Kirby Research Center for Functional Brain Imaging at the Kennedy Krieger Institute on a 3T Philips Achieva MRI magnet with a 32-channel head coil. High resolution 3D MPRAGE images were acquired with the following parameters: 170 axial slices, 1 mm^3^ voxels, FOV = 256 × 240 × 240 mm, TR = 6.8 ms, and TE = 3.2 ms. Resting state blood-oxygen-level-dependent (BOLD) sensitive images were obtained using a gradient echo EPI sequence with the following parameters: 35 axial slices, 3 mm^3^ voxels, 210 volumes/run, FOV = 240 × 240 mm, flip angle = 75°, phase encoding directio*n* = AP, TR = 2000 ms, TE = 30 ms. Twelve participants completed one run of the BOLD sequence while the remaining 33 individuals completed two BOLD sequence runs.

### 2.4 MR data analysis

#### 2.4.1 Preprocessing

**Figure 1:**
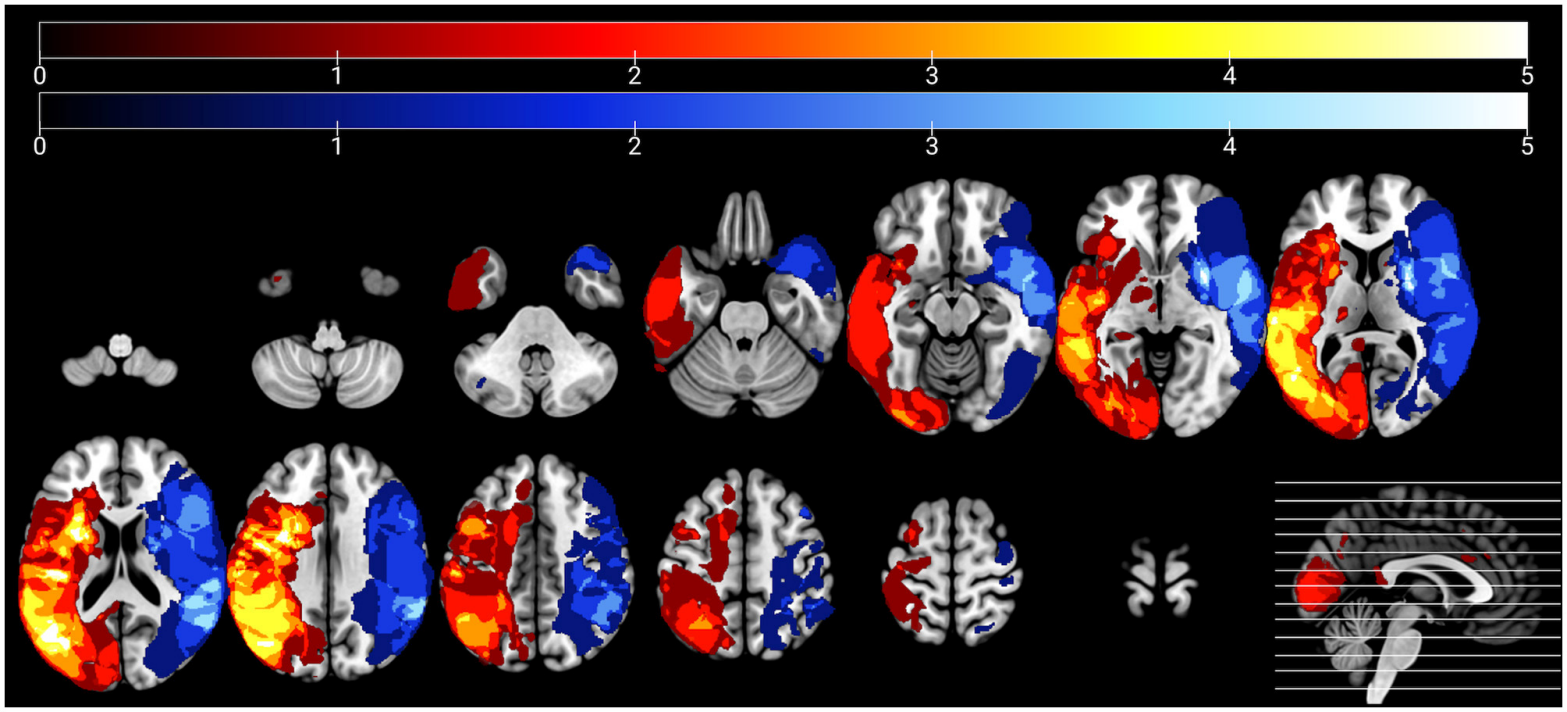
Lesion overlays of participants with left hemisphere damage (*n* = 28) and right hemisphere damage (*n* = 17) are shown in warm and cool colors, respectively. Maximum overlap of each overlay is five participants per group.

Using MRIcron (https://www.nitrc.org/projects/mricron), two trained members of the research team blinded to the behavioral data (CS and JS) manually traced lesioned tissue slice-by-slice in native space in the axial plane of each participant's T1-weighted image (see Figure 1 for lesion overlays for each group). A third author (EM) reviewed and edited lesion tracings as needed. Lesion maps (in which lesioned voxels were retained) were binarized and subsequently incorporated into preprocessing and used to calculate total lesion volume, cerebral damage laterality indices, and the percentage of damaged tissue within regions of interest (ROIs) for the rs-FC analyses.

MR data were processed in SPM12 (https://www.fil.ion.ucl.ac.uk/spm/software/spm12/) using standard routines with some additional steps to account for stroke lesions. After the first five functional volumes were discarded, differences in the timing of slice acquisition in the functional scans were corrected via the slice timing correction routine with reference to the middle slice. Next, realignment of the functional scans to the mean image was performed via 4th degree B-spline interpolation, and the T1-weighted image, lesion map, and all functional volumes were coregistered. The Clinical Toolbox (61) was used to warp each participant's T1-weighted image and lesion map to a standard template of neurologically-healthy older adults. Before completing additional preprocessing steps, the lead author (EM) checked normalization accuracy by assessing the alignment of normalized data to the template image using SPM12's CheckReg function. The T1-weighted images were subsequently segmented into gray matter, white matter, and cerebrospinal fluid using SPM12's tissue probability maps. After this step, we visually assessed the subject-specific segmentations to verify that the lesion was segmented into the CSF mask and out of the gray matter mask. Functional data were then spatially smoothed using a 6 mm FWHM kernel. Given that rs-fMRI data are particularly susceptible to head motion (62), the Artifact Detection Tools toolbox (ART; https://www.nitrc.org/projects/artifact_detect) was used to identify motion outliers. Volumes were flagged as poor quality in ART if the global signal value was three standard deviations away from the mean or higher, linear motion displacement was 0.5 mm or higher between volumes, and/or the rotation was ≥0.02 radians.

Prior to functional connectivity analysis, we completed additional analyses using normalized lesion data. First, we defined and extracted ROIs from each resting state network of interest. For the DMN, DAN, and SN, we used ROIs stored within the CONN Toolbox (63) that were defined based on an independent component analysis of resting data from 497 healthy subjects. Language network ROIs were defined based on prior language meta-analyses and systematic reviews in neurologically healthy individuals and PWA (36, 44, 64–66) and extracted from the Johns Hopkins University atlas (67). We resampled the ROIs to the resolution of the normalized lesion maps using FreeSurfer's “mri vol2vol” function (https://surfer.nmr.mgh.harvard.edu/fswiki/mri_vol2vol) and then warped the resampled ROIs to the older adult template using FSL's FMRIB's Linear Image Registration Tool (https://fsl.fmrib.ox.ac.uk/fsl/fslwiki/FLIRT).

Next, using a bespoke MATLAB script based on MarsBaR routines (68), we intersected each participant's normalized lesion map with network ROIs. This resulted in a set of ROIs for every participant that included only their spared ROI tissue. Finally, we calculated the total normalized lesion volume, separate LH and RH damage volumes, and the percent damage to ROIs and networks for each individual. We generated a cerebral damage laterality index for each participant by subtracting the number of damaged RH voxels from the number of damaged LH voxels and dividing that value by the subject's lesion map volume. Based on this calculation, a laterality index of 1 corresponds to a LH lesion with no RH damage, and a laterality index of −1 corresponds to a RH lesion with no LH damage. As reported in Table 1, the LHD and RHD groups did not significantly differ in total lesion volume, the absolute value of laterality indices, or the number of participants whose strokes occurred within the MCA territory or incurred cortical damage (*p* > 0.05 across tests) (Table 1). See Supplementary Table 2 for additional stroke location information for each participant. Individual ROI percent damage values are included in Supplementary Tables 3–7.

#### 2.4.2 Functional connectivity and statistical analyses

Functional connectivity and statistical analyses were completed using the CONN Toolbox, version 22 (69). First, we imported the SPM preprocessed data into the CONN Toolbox and completed denoising using the CompCor method (70). Next, we applied a bandpass filter of 0.008–0.09 Hz and removed linear trends in the data. We then performed a linear regression analysis on noise sources including white matter and CSF signals, the six rigid body head motion parameters extracted from ART, motion outliers from ART, and the main effect of rest. For the first-level analysis, we conducted a weighted General Linear Model (GLM) with bivariate ROI-to-ROI correlations for each participant and network. For this analysis, we imported the subject-specific network ROIs into CONN to use as seeds. This approach ensured that only spared tissue was seeded for functional connectivity analysis. Some network ROIs were completely damaged for certain participants (see Supplementary Table 1). For these individuals, we seeded the original network ROIs after verifying that the lesion was properly segmented from the gray matter mask. This was done to account for the lesion but maintain the same ROI matrix structure across participants.

At the second level, we conducted three sets of analyses within CONN. First, we performed two-sample *t*-tests on the functional connections within the four intrinsic networks, with each network examined separately to understand how intrinsic connectivity of these networks differed between groups in the absence of its relationship with picture description metrics. Language processing is highly left-lateralized (unlike cognitive processes mediated by other networks), and many participants had middle cerebral artery (MCA) strokes, making language network regions in both hemispheres highly susceptible to damage in both patient groups. As such, for this initial analysis, we split the language network by hemisphere to allow us to evaluate more clearly the impact of stroke on intrinsic connectivity of the ipsilesional hemisphere. Mean-centered picture description informativeness measures and total lesion volume were added as second-level covariates for the two main Aim 2 analyses. To address Aim 2a, we conducted within-group regression analyses to determine which connections were associated with picture description metrics separately for each group. To address Aim 2b, we conducted one-way ANCOVAs to determine between-group differences in the relationship between rs-FC and picture description metrics. All 43 ROIs were included in each of the main Aim 2 analyses. This approach allowed us to determine whether the relationships between picture description measures and rs-FC were linked to within vs. between-network functional connections. For each analysis, we interrogated rs-FC using the multivariate approach described in Jafri et al. (71), in which clusters of ROIs are first defined via a data-driven hierarchical clustering procedure (based on functional similarity) and then a multivariate GLM analysis is applied for all connections within each cluster of connections. For each analysis, we applied an MVPA omnibus test-derived cluster-level FDR correction at *q* < 0.05 with a connection-level threshold of *p* < 0.05 (uncorrected). The FDR-corrected cluster level *q* value reflects the expected proportion of false discoveries among connection pairs with larger or similar effects across the entire set of connections.

## 3 Results

### 3.1 Group differences in picture description informativeness measures

**Figure 2:**
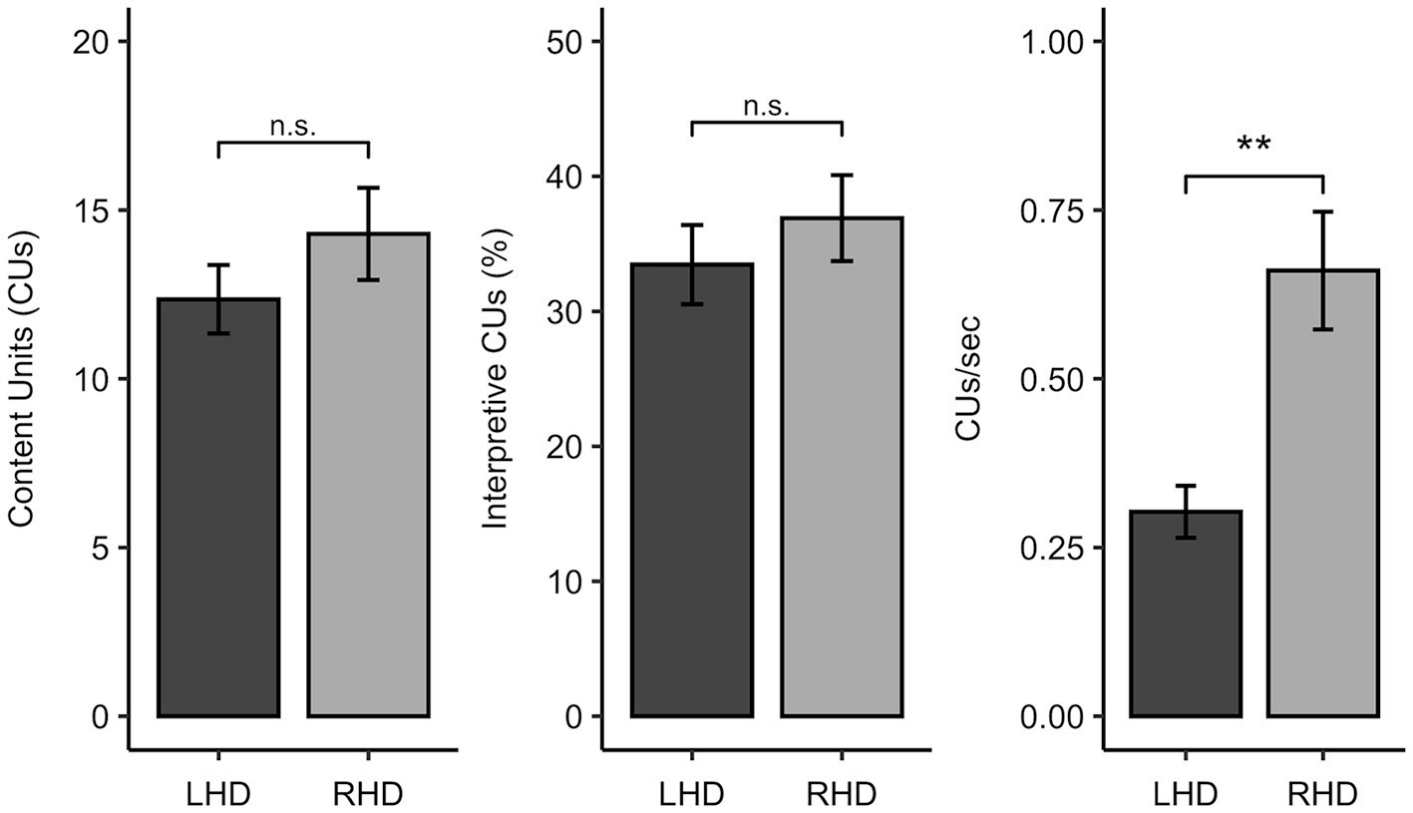
Comparison of participants with left hemisphere damage (LHD) and right hemisphere damage (RHD) in Cookie Theft content unit (CU) measures. ** < 0.01, n.s., not significant.

The LHD and RHD groups did not significantly differ in the total number of CUs produced (*W* = 273.0, *p* = 0.418, *q* = 0.582) or in the percentage of interpretative CUs (*W* = 262.0, *p* = 0.582, *q* = 0.582). In contrast, participants with LHD produced significantly fewer CUs/second than individuals with RHD (*W* = 99.0, *p* = 0.001, *q* = 0.004) (Figure 2).

To further characterize the picture description skills of the sample, we also classified each participant as impaired or unimpaired on each measure of interest. Eleven of the 28 individuals with LHD (39% of the group) had CU counts that fell below normal limits (indicative of impairment), while three of the 17 participants with RHD (18% of the group) were impaired on this measure. Thirteen out of 28 individuals with LHD (46%) and six out of 17 people with RHD (35%) had percent interpretative CU scores that were at least 1.5 standard deviations below the control sample from Myers (24). While only two of the 17 individuals in the RHD sample (12%) demonstrated slowed efficiency of CU production, exactly half of the LHD sample produced fewer than normal CUs/second. Consistent with the comparisons of these measures as continuous variables, Fisher's exact tests revealed that the number of impaired vs. unimpaired participants significantly differed between the LHD and RHD groups for CUs/second (OR = 0.139, *p* = 0.012, *q* = 0.035) but not total CUs (OR = 0.339, *p* = 0.188, *q* = 0.282) or percentage of interpretive CUs (OR = 0.481, *p* = 0.356, *q* = 0.356).

### 3.2 Functional connectivity findings

**Figure 3:**
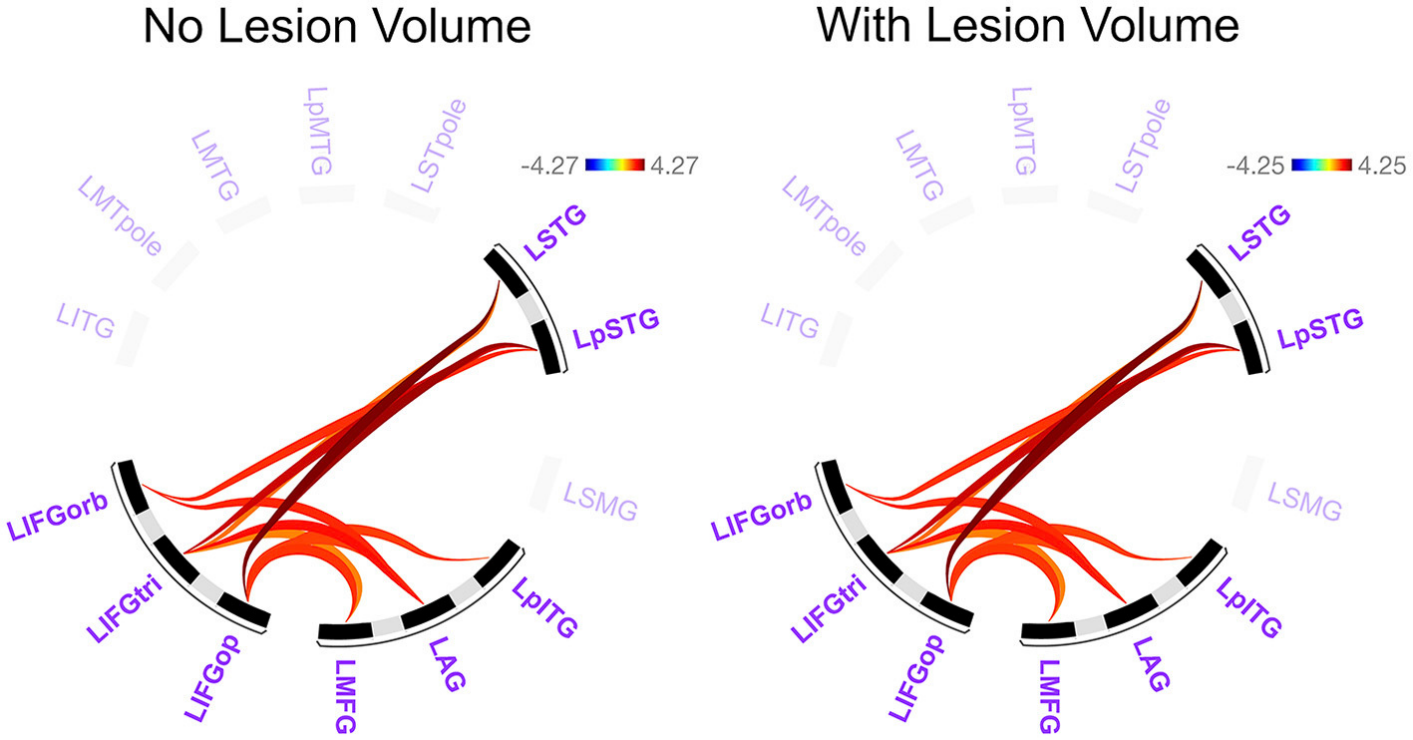
Significant differences between groups in left language network connectivity. Warmer colors indicate connections that are significantly stronger in individuals with right hemisphere compared to left hemisphere damage. Region names included in significant connections are in bold font. Language network regions are in purple font. Region labels: AG, angular gyrus; IFGop, inferior frontal gyrus, pars opercularis; IFGorb, IFG, pars orbitalis; IFGtri, IFG, pars triangularis; ITG, inferior temporal gyrus; L, left; MFG, middle frontal gyrus; MTG, middle temporal gyrus; MTpole, middle temporal pole; p, posterior; SMG, supramarginal gyrus; STG, superior temporal gyrus; STpole, superior temporal pole.

Before addressing our main Aim 2 objectives, we compared rs-FC for each network separately to gain a better understanding of the potential differences LH vs. RH lesions had on intrinsic network connectivity. There were no significant differences between groups in rs-FC of the DAN, DMN, right LN, or SN (*p* > 0.05). As shown in Figure 3 and Table 2, left intra-hemispheric connectivity of the LN was significantly greater in the RHD vs. LHD groups for the first cluster (of 14) involving connections between the mid and posterior parts of left STG (LSTG) and all three parts of the left IFG (LIFG) [*F*_(2, 42)_ = 8.23, *p* < 0.001, *q* = 0.013]. Stronger rs-FC for patients with RHD vs. LHD was also noted for a cluster involving connections between the left AG (LAG), left middle frontal gyrus (LMFG), left inferior posterior temporal gyrus (LpITG) and all three parts of LIFG [*F*_(2, 42)_ = 6.17, *p* = 0.004, *q* = 0.031]. Controlling for total lesion volume slightly altered the strength of the statistics but not the clusters or connections that differed between groups (see Figure 3 and Table 2). An analysis conducted on the entire set of LN ROIs from both hemispheres was not significant at the cluster-level FDR threshold of *q* < 0.05, but uncorrected results (at *p* < 0.001) largely reflected the left LN only analysis. Specifically, as shown in Supplementary Figure 1 and Supplementary Table 8, stronger rs-FC for patients with RHD vs. LHD was noted for connections involving the three parts of LIFG and their functional synchrony with the left superior temporal pole (LSTpole), mid LSTG, and LpSTG as well as one inter-hemispheric connection involving RSTG.

**Table 2:** Differences between groups in left language network connectivity.

**Analysis unit**	**Test statistic**	***p-*value**	***q-*value (FDR correction)**
**Without controlling for lesion volume**			
Cluster 1 (of 14)	*F*_(2, 42)_ = 8.23	< 0.001	0.013
LIFGop-LSTG	*t*_43_ = 4.27	< 0.001	0.001
LIFGop-LpSTG	*t*_43_ = 3.99	< 0.001	0.002
LIFGtri-LpSTG	*t*_43_ = 3.60	< 0.001	0.011
LIFGorb-LpSTG	*t*_43_ = 2.80	0.008	0.050
LIFGtri-LSTG	*t*_43_ = 2.24	0.031	0.080
Cluster 2 (of 14)	*F*_(2, 42)_ = 6.17	0.004	0.031
LIFGtri-LAG	*t*_43_ = 3.06	0.004	0.025
LIFGop-LMFG	*t*_43_ = 2.77	0.008	0.026
LIFGop-LpITG	*t*_43_ = 2.68	0.010	0.026
LIFGop-LAG	*t*_43_ = 2.62	0.012	0.026
LIFGorb-LAG	*t*_43_ = 2.88	0.006	0.050
LIFGtri-LMFG	*t*_43_ = 2.11	0.040	0.080
**Controlling for lesion volume**			
Cluster 1 (of 14)	*F*_(2, 41)_ = 8.24	< 0.001	0.014
LIFGop-LSTG	*t*_42_ = 4.25	< 0.001	0.002
LIFGop-LpSTG	*t*_42_ = 3.98	< 0.001	0.002
LIFGtri-LpSTG	*t*_42_ = 3.58	< 0.001	0.012
LIFGorb-LpSTG	*t*_42_ = 2.77	0.008	0.054
LIFGtri-LSTG	*t*_42_ = 2.25	0.029	0.086
Cluster 2 (of 14)	*F*_(2, 41)_ = 8.24	0.005	0.034
LIFGtri-LAG	*t*_42_ = 3.03	0.004	0.027
LIFGop-LMFG	*t*_42_ = 2.73	0.009	0.029
LIFGop-LpITG	*t*_42_ = 2.63	0.012	0.029
LIFGop-LAG	*t*_42_ = 2.58	0.013	0.029
LIFGorb-LAG	*t*_42_ = 2.83	0.007	0.054
LIFGtri-LMFG	*t*_42_ = 2.11	0.041	0.086

Positive t-values indicate connections that are significantly stronger in individuals with right hemisphere compared to left hemisphere damage. AG, angular gyrus; IFGop, inferior frontal gyrus, pars opercularis; IFGorb, IFG, pars orbitalis; IFGtri, IFG, pars triangularis; ITG, inferior temporal gyrus; L, left; MFG, middle frontal gyrus; p, posterior; STG, superior temporal gyrus.

#### 3.2.1 Aim 2a: within-group associations between rs-FC and picture description measures

**Figure 4:**
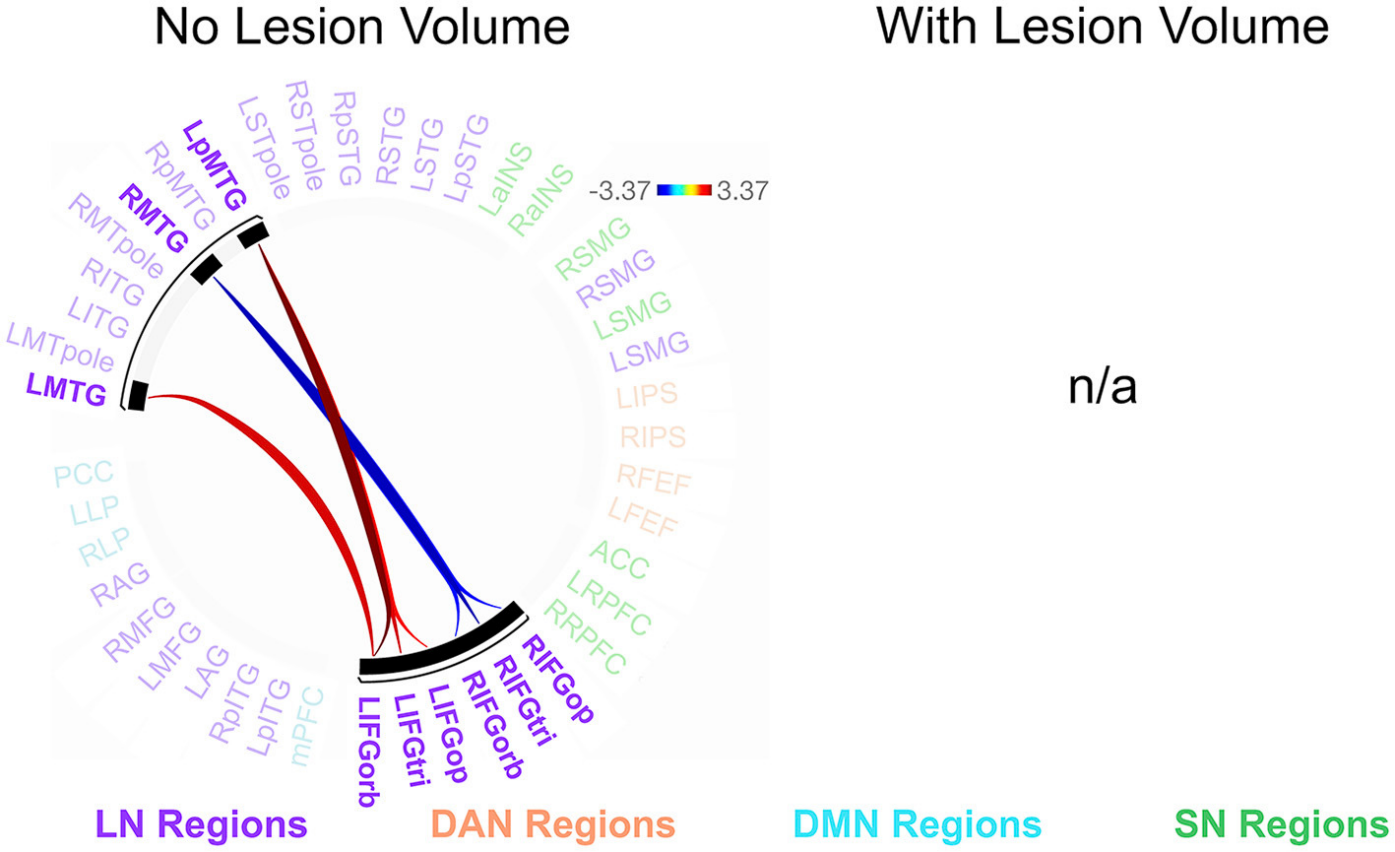
Relationships between network connectivity and CUs/second in the left hemisphere stroke group. Warm-colored lines indicate a relationship between stronger connectivity and a greater number of content units (CUs) produced per second in individuals with left hemisphere damage. Cool-colored lines indicate a relationship between weaker connectivity and higher CUs/second. Lesion volume control was not performed within the CONN Toolbox due to an incomplete model with two covariate variables and instead investigated outside the CONN Toolbox (see section 3.2.2). Region names included in significant connections are in bold font. LN, language network, DAN, dorsal attention network, DMN, default mode network, SN, salience network. Region labels: ACC, anterior cingulate cortex; AG, angular gyrus; a, anterior; FEF, frontal eye field; IFGop, inferior frontal gyrus, pars opercularis; IFGorb, IFG, pars orbitalis; IFGtri, IFG, pars triangularis; ITG, inferior temporal gyrus; IPS, intraparietal sulcus; L, left; LP, lateral parietal cortex; MFG, middle frontal gyrus; MPFC, medial prefrontal cortex; MTG, middle temporal gyrus; MTpole, middle temporal pole; p, posterior; PCC, posterior cingulate cortex; R, right; RPFC, rostral prefrontal cortex; SMG, supramarginal gyrus; STG, superior temporal gyrus; STpole, superior temporal pole.

We found no significant relationships between rs-FC and picture description measures within the RHD group. In the LHD group, the total number of CUs and percentage of interpretive CUs were not significantly related to rs-FC after the cluster-level FDR correction. In contrast, we found that CUs/second was significantly associated with the first cluster (of 28) of connections in participants with LHD [*F*_(2, 25)_ = 8.49, *p* = 0.002, *q* = 0.043]. Specifically, a higher number of CUs/second was significantly associated with stronger rs-FC between all three parts of LIFG and the left posterior middle temporal gyrus (LpMTG) but weaker rs-FC between all three parts of right IFG (RIFG) and the right middle temporal gyrus (RMTG) (see Figure 4 and Table 3). In other words, better communication efficiency of salient scene content was related to stronger left and weaker right frontotemporal connectivity in the LHD group.

**Table 3:** Relationships between network connectivity and CUs/second in the LHD group.

**Analysis unit**	**Test statistic**	***p-*value**	***q-*value (FDR correction)**
**Without controlling for lesion volume**			
Cluster 1 (of 28)	*F*_(2, 25)_ = 8.49	0.002	0.043
RIFGtri-RMTG	*t*_26_ = −3.10	0.005	0.058
LIFGorb-LpMTG	*t*_26_ = 3.37	0.002	0.089
LIFGorb-LMTG	*t*_26_ = 2.96	0.007	0.091
LIFGtri-LpMTG	*t*_26_ = 2.96	0.007	0.137
RIFGop-RMTG	*t*_26_ = −2.36	0.0258	0.272
RIFGorb-RMTG	*t*_26_ = −2.22	0.0354	0.539
LIFGop-LpMTG	*t*_26_ = 2.26	0.0324	0.703

Positive t-values indicate a relationship between stronger connectivity and a greater number of content units (CUs) produced per second in individuals with left hemisphere damage (LHD). Negative t values indicate a relationship between weaker connectivity and higher CUs/second. Lesion volume control was not performed within the CONN Toolbox due to an incomplete model with two covariates. IFGop, inferior frontal gyrus, pars opercularis; IFGorb, IFG, pars orbitalis; IFGtri, IFG, pars triangularis; L, left; MTG, middle temporal gyrus; p, posterior; R, right.

To test whether the directionality of these findings was related to overall lesion volume or damage to LIFG specifically, we conducted four follow-up Spearman correlations between lesion metrics (i.e., total lesion volume, percent damage to LIFG) and averaged effect sizes involving left or right intra-hemispheric connections. Overall lesion volume was not significantly associated with effect sizes reflecting the relationship between CUs/second and left (*r* = −0.215, *p* = 0.269, *q* = 0.388) or right (*r* = −0.158, *p* = 0.421, *q* = 0.421) frontotemporal connections. Similarly, the association between LIFG damage and effect sizes between CUs/second and the RIFG-RMTG connection was not significant before or after FDR correction (*r* = −0.207, *p* = 0.291, *q* = 0.388). In contrast, greater LIFG damage was significantly associated with smaller effect sizes between CUs/second and left frontotemporal connections, although this finding did not survive correction for multiple comparisons (*r* = –.384, *p* = 0.043, *q* = 0.174).

#### 3.2.2 Aim 2b: between-group differences in the relationship between rs-FC and picture description measures

**Figure 5:**
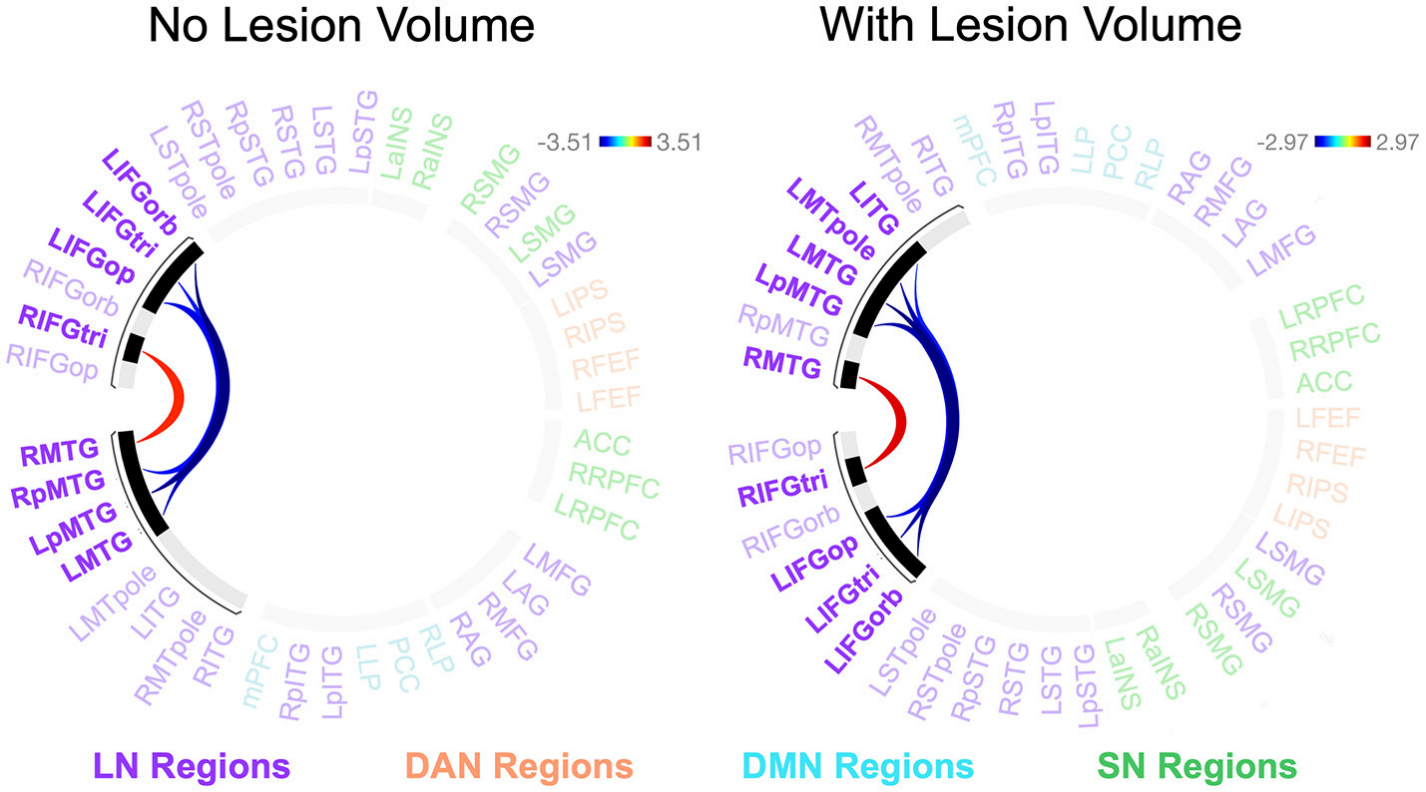
Between-group differences in the relationship between network connectivity and CUs/second. Cool-colored lines indicate a weaker relationship between connectivity and CUs/second in individuals with right hemisphere compared to left hemisphere damage whereas the opposite is true of warm-colored lines. Region names included in significant connections are in bold font. LN, language network, DAN, dorsal attention network, DMN, default mode network, SN, salience network. Region labels: ACC, anterior cingulate cortex; AG, angular gyrus; a, anterior; FEF, frontal eye field; IFGop, inferior frontal gyrus, pars opercularis; IFGorb, IFG, pars orbitalis; IFGtri, IFG, pars triangularis; ITG, inferior temporal gyrus; IPS, intraparietal sulcus; L, left; LP, lateral parietal cortex; MFG, middle frontal gyrus; MPFC, medial prefrontal cortex; MTG, middle temporal gyrus; MTpole, middle temporal pole; p, posterior; PCC, posterior cingulate cortex; R, right; RPFC, rostral prefrontal cortex; SMG, supramarginal gyrus; STG, superior temporal gyrus; STpole, superior temporal pole.

Participants with LHD and RHD did not significantly differ in the relationships between rs-FC and the total number of CUs or percentage of interpretive CUs, but there was a significant difference between groups in the relationship between CUs/second and rs-FC for one cluster (of 45) [*F*_(2, 40)_ = 10.50, *p* < 0.001, *q* = 0.010]. In general, the relationship between CUs/second and left frontotemporal connectivity was significantly weaker in patients with RHD compared to the LHD group (see Figure 5 and Table 4). As the only exception, the relationship between CUs/second and rs-FC between RIFG, pars triangularis (RIFGtri) and RMTG was significantly stronger in the RHD group than individuals with LHD. Participants in the LHD group also exhibited a stronger relationship between CUs/second and one interhemispheric connection between LIFG, pars opercularis (LIFGop) and RpMTG.

**Table 4:** Between-group differences in the relationship between network connectivity and CUs/second.

**Analysis unit**	**Test statistic**	***p-*value**	***q-*value (FDR correction)**
**Without controlling for lesion volume**			
Cluster 1 (of 45)	*F*_(2, 40)_ = 10.50	< 0.001	0.010
LIFGorb-LpMTG	*t*_41_ = −3.51	0.001	0.047
LIFGop-LpMTG	*t*_41_ = −2.79	0.008	0.160
LIFGtri-LpMTG	*t*_41_ = −2.44	0.019	0.160
LIFGorb-LMTG	*t*_41_ = −3.07	0.004	0.160
LIFGop-RpMTG	*t*_41_ = −2.52	0.016	0.170
LIFGop-LMTG	*t*_41_ = −2.47	0.018	0.272
RIFGtri-RMTG	*t*_41_ = 2.35	0.024	0.277
**Controlling for lesion volume**			
Cluster 1 (of 45)	*F*_(2, 39)_ = 8.18	0.001	0.049
LIFGorb-LMTG	*t*_40_ = −2.97	0.005	0.160
LIFGorb-LpMTG	*t*_40_ = −2.74	0.009	0.160
LIFGorb-LMTpole	*t*_40_ = −2.65	0.011	0.160
LIFGtri-LMTpole	*t*_40_ = −2.70	0.010	0.213
RIFGtri-RMTG	*t*_40_ = 2.41	0.020	0.215
LIFGop-LpMTG	*t*_40_ = −2.38	0.022	0.271
LIFGop-LMTpole	*t*_40_ = −2.34	0.024	0.271
LIFGop-LMTG	*t*_40_ = −2.32	0.026	0.271
LIFGop-LITG	*t*_40_ = −2.03	0.049	0.372
LIFGtri-LpMTG	*t*_40_ = −2.07	0.045	0.401

Negative t values indicate a weaker relationship between connectivity and CUs/second in individuals with right hemisphere compared to left hemisphere damage whereas the opposite is true of positive t values. IFGop, inferior frontal gyrus, pars opercularis; IFGorb, IFG, pars orbitalis; IFGtri, IFG, pars triangularis; ITG, inferior temporal gyrus; L, left; MTG, middle temporal gyrus; MTpole, middle temporal pole; p, posterior; R, right.

When controlling for total lesion volume, the first cluster (of 45) was still significant [*F*_(2, 39)_ = 8.18, *p* = 0.001, *q* = 0.049], and six of the seven significant connections included in this cluster were the same as those in the analysis without the lesion volume covariate (Figure 5 and Table 4). While the relationship difference involving LIFGop-RpMTG was no longer significant, additional significant connections identified by this analysis included LIFGop to left ITG (LITG) and the connectivity between all three parts of LIFG and the left middle temporal pole (LMTpole).

## 4 Discussion

In this study, we investigated differences between individuals with early chronic left vs. right hemisphere stroke in their ability to produce meaningful content during picture description and examined the resting state substrates of within- and between-group differences in those abilities. Despite the ever-growing body of discourse neuroimaging literature, to our knowledge, this investigation is the first to report direct comparisons between individuals with LHD and RHD with regards to relationships between brain function and discourse skills. We address each set of findings in greater detail below.

### 4.1 Comparisons between individuals with LHD vs. RHD in picture description informativeness

Before turning to the functional connectivity results, we first address the behavioral findings. Contrary to our predictions, we found that our participant groups did not differ in the total number of CUs or in the percentage of interpretative CUs produced during the Cookie Theft picture description task. Similar to our findings, no group effects were reported for total Cookie Theft CUs or interpretive CUs by Tompkins et al. (22) or for lexical informativeness of picture sequence descriptions by Schneider and colleagues (15, 16), meaning that individuals with LH and RH chronic stroke in their studies did not statistically differ from controls or each other in the production of meaningful narrative content. In contrast to our findings, Agis et al. (9) reported that patients with acute LH stroke produced statistically fewer CUs than patients with acute RH stroke (although group means did not vastly differ) and that patients in both groups produced fewer CUs than neurologically healthy controls. These contrasting findings may be due to differences between studies in the stroke chronicity and overall deficit severity of the participants, an explanation Schneider et al. (15, 16) also proposed. Deficits in stroke survivors are typically most pronounced in the acute stroke phase. Thus, it is likely that patients in both groups in Agis et al. (9) had more severe overall language impairments than individuals in either chronic stroke study and that the characteristic challenges individuals with aphasia have in word retrieval most greatly impacted the participants with LH stroke in Agis et al. (9).

In general, deficit severity is an important factor to consider when interpreting discourse production abilities in individuals with acquired neurological disorders. While the LH stroke group in Schneider et al. (16) had lower cognitive scores and naming abilities than healthy controls, only one individual presented with a mild aphasia. Their RH stroke group did not differ from controls on neuropsychological assessments, which suggests their patient groups consisted of individuals with only mild impairments. Like Schneider et al. (15, 16), our LHD group included individuals with mild or negligible linguistic deficits, at least according to the CU measures analyzed in this paper. Note that such participants may have struggled with a different aspect of linguistic processing (e.g., syntactic production, phonological retrieval) that we did not capture with our measures. Unlike Schneider et al. (15, 16), we also included participants with aphasia who had impaired CU production (as denoted by asterisks next to individual scores in Supplementary Table 1). Most of these individuals produced either phonemic paraphasias (e.g., “overflilling” for *overflowing*), neologisms (e.g., “donking” for *stealing*), or semantic errors (e.g., “chair” for *stool*; empty speech) that reduced their CU scores. Deficits in discourse informativeness may be attributed to PWAs' anomia, which might be exacerbated during discourse production due to heightened competition between activated lexical targets in connected speech (72).

The consistency of discourse production impairments and their relation to global cognitive skills in RH stroke is more difficult to quantify, partially because of the relative lack of these studies in RHD (73), especially compared to LH stroke. Furthermore, while individuals with RHD can exhibit word selection deficits, their errors may be less blatant than the paraphasias produced by PWA and less distinguishable from the types of errors made by older adults with no history of stroke (74). In RH stroke, word choice errors may arise due to either insufficient activation of semantically distant word meanings (per the coarse coding deficit hypothesis) (75–77) or reduced inhibition of competing, inappropriate semantic alternatives (per the suppression deficit hypothesis) (78, 79). In our study, only one of the three RH stroke survivors with impaired CU scores produced an obvious semantic error (i.e., “chair” for *stool*), but all three participants' samples lacked specificity that impacted their CU scores. For example, one individual (P40) referenced a “number of disasters” occurring in the scene but did not specify that the boy was about to fall off the stool or that the sink was overflowing. Interestingly, a higher percentage of the RHD group had impaired interpretative CU scores (*n* = 6, 35% of the group), but this small percentage was still unexpected based on prior literature [e.g., (23, 24)] and in comparison to the LHD group. It is likely other researchers studied patients with larger lesions with persisting deficits compared to our participants, as we enrolled all consenting RH stroke patients in the acute phase, irrespective of whether they had noticeable deficits. Unfortunately, it is unknown whether reductions in CUs and interpretive CU production were associated with deficits in other cognitive domains (e.g., executive functions, attention) given that our testing battery did not include a comprehensive cognitive assessment, a point that we return to in “Study limitations.”

Unlike the first two picture description measures, CUs/second differed between groups, both in terms of raw values and in the number of individuals classified as impaired within each group. As a group, individuals with LHD produced significantly fewer CUs/second than participants with RHD. Half of the LHD group exhibited impaired performance on this measure whereas only two participants with RHD had scores that fell below normal limits. In Agis et al. (9), both LHD and RHD groups had significantly lower CUs/minute scores than controls but the two patient groups did not differ from each other, which perhaps again reflects the stroke chronicity and/or severity differences between our sample and that of Agis et al. (9). The discrepancy between the number of participants in the LHD group who were impaired on total CUs vs. CUs/second may reflect lingering deficits in processing speed in some individuals in this group. Individuals with anomic (80) and recovered (81) aphasia may require twice the amount of time as neurologically healthy adults to retrieve the names of objects or actions in isolation. In connected speech, words need to be appropriately embedded into clauses which in turn need to be combined to form sentences that must collectively appropriately and quickly describe a pictured scene or capture a remembered event or procedure. Thus, it stands to reason that communication efficiency as captured in our study would be impaired in a large number of individuals in the LHD group.

### 4.2 Relationships between language network connectivity and efficient communication of picture content

Like the behavioral results, there were no between-group differences in the relationship between rs-FC and the total number of CUs or percentage of interpretative CUs. We also found no significant associations between rs-FC for these two measures within either group nor between rs-FC and CUs/second for participants in the RHD group. The lack of significant within-group results for the total number of CUs and percentage of interpretative CUs may be due in part to reduced statistical power with the smaller group sizes, particularly for the RHD group. Larger samples may be needed to identify nuances in the relationships between rs-FC and these measures, particularly since most participants with RHD had informativeness scores that fell within normal limits. It may also be that of these three measures, CUs/second is the most sensitive metric to capture the functional consequences of stroke in individuals with LHD who are most likely to struggle with rapid communication of scene content.

Perhaps because of this, the most thought-provoking resting state findings pertained to relationships between rs-FC and CUs/second. In participants with LHD, higher CUs/second scores were associated with stronger left and weaker right intra-hemispheric frontotemporal rs-FC of the LN. In accordance with the within-group results, the relationship between rs-FC and left frontotemporal connectivity was significantly stronger in individuals with LHD compared to participants in the RHD group. As expected, the LHD group had weaker left LN intra-hemispheric connectivity than the RHD group (see Table 2), but none of the connections that were inherently weaker in the LHD group were ones that correlated with CUs/second. As such, the connectivity findings for CUs/second do not merely reflect inherent differences between groups in intrinsic connectivity but instead accord with previous meta-analyses and systematic reviews indicating that when possible, PWA utilize residual tissue within the left hemisphere LN for language processing (65, 66, 82, 83). When significant brain-behavior relationships involving left intra-hemispheric connections have been reported in PWA, the consistent finding across task-based and resting state studies is that individuals who have stronger left intra-hemispheric connectivity also have better language skills [e.g., (84–89)].

In contrast, findings regarding RH activity and connectivity remain mixed. Increased activity in RH homologs of damaged LH brain regions is believed to be a marker of transcallosal disinhibition (90) and potentially maladaptive for language recovery, a theory supported by certain cross-sectional investigations [e.g., (91–93)] and language treatment studies [e.g., (94–96)]. In other studies, however, an upregulation of RH activity has been associated with better language performance [e.g., (82, 97, 98)] and pre- to post-treatment language gains [e.g., (99–103)]. While still sparse, cross-sectional studies that report findings regarding relationships between language and right intra-hemispheric connectivity also suggest a potential beneficial role of increased connectivity of RH connections for people with chronic aphasia. For example, using dynamic causal modeling, Teki et al. (104) found that better semantic abilities were related to greater effective connectivity from RSTG to the right auditory cortex in individuals with chronic aphasia. Similarly, in Meier et al. (105), participants with aphasia who demonstrated better performance (i.e., longer reaction times, higher accuracy) during a semantic feature judgment fMRI task also exhibited stronger effective connectivity of a group of connections that included intra-hemispheric connections between RIFGtri, RMTG, and RITG. It seems likely that there is individual variability in the role of the RH in recovery; some people (perhaps those with the largest LH strokes) depend on the RH to assume language functions, while in those with better function of the LH, RH activation may be maladaptive.

In our study, it is important to note that the significant results for CUs/second were restricted to synchrony between either LIFG or RIFG and temporal lobe regions. As such, the inverse directionality of the left and right intra-hemispheric connectivity findings may reflect a more precise distinction between the roles of LIFG, RIFG, and specific frontotemporal connections for language processing rather than a more general hemispheric difference in LH stroke recovery. Increased activation and/or connectivity of parts of LIFG have often been linked to better language skills and treatment outcomes in PWA (106–112). Specific to discourse production, Alyahya et al. (26) found that reduced content word accuracy and appropriateness across different discourse genres were associated with widespread left frontal damage, including to LIFGop, LIFGtri, and LIFG, pars orbitalis (LIFGorb) (as well as LMFG) in 46 individuals with chronic aphasia. Keser et al. (27) reported trends between the total number of CUs produced during picture description by individuals either six or 12 months post-stroke and the microstructural integrity of the left arcuate, left inferior longitudinal, and left inferior fronto-occipital fasciculi. Association fibers belonging to the left arcuate and inferior fronto-occipital fasciculi connect portions of LIFG to the posterior temporal cortex (113, 114) and correspond to the functional connections between LIFG and the mid to posterior temporal regions implicated for CUs/second in our study. In healthy individuals, Humphreys and Lambon Ralph (115) found that both LIFG and LpMTG are sensitive to semantic (but not visuospatial) and hard (but not easy) tasks and demonstrated very similar patterns of connectivity when seeded. In PWA, reduced connectivity between LIFG and LpMTG has been linked to impaired lexical-semantic processing and reduced semantic control, particularly for individuals with LIFG lesions (85, 87).

When accounting for total lesion volume, the relationships between CUs/second and connections from portions of LIFG to other parts of the left temporal lobe (i.e., LITG, LMTG, and LMTpole) were also stronger in the LHD group than in individuals with RHD. The mid MTG and ITG are believed to act as convergence zones that store object and event knowledge received from the primary sensory cortices (116) or may be regions that play a central role in combinatorial semantics (34). In the hub-and-spoke model of semantic cognition (117), the anterior temporal lobe is considered a modality-invariant hub where modality-specific information received from other parts of the brain is stored and processed. While the specific functions of these various regions remain disputed between semantic models, these collective findings are consistent with the notion that LIFG, the anterior temporal lobe, and the mid to posterior left temporal cortex are essential, highly connected hubs within the semantic network (115, 117–119) and when spared, are recruited for semantic tasks by individuals with LHD.

While beneficial RIFG activity has also been reported—and sometimes within the same sample as positive LIFG findings [e.g., (108, 110, 120)]—the *necessity* of RIFG vs. LIFG engagement across different language tasks is still an open question. Meta-analytic findings indicate that both PWA and controls activate RIFG across a variety of language tasks, but that PWA may engage portions of RIFG to a greater extent than their neurologically healthy peers (65, 82, 83). However, epiphenomenal RH activity that does not contribute to language performance has been suggested in prior literature (121–123). Non-invasive brain stimulation studies provide more causal conclusions pertaining to the roles of LIFG vs. RIFG for language processing. Inhibitory stimulation of RIFG and/or excitatory stimulation of LIFG often results in greater left-lateralized activity which coincides with improvements in production tasks such as picture naming and word fluency [see (124–128) for review], effects which in some studies [e.g., (126)] are specific to individuals with LIFG lesions.

Relatedly, it has been suggested that heightened RH activity (or connectivity) may be beneficial for language processing in PWA with large LH lesions due to the lack of viable remaining LH tissue (90, 129). Supporting this hypothesis, RIFG activity in past activation studies has often been associated with better language skills in individuals with LIFG damage [e.g., (98, 130, 131)]. In our study, however, we found that the effect size for the relationship between CUs/second and right frontotemporal connectivity within the LHD group was neither positively nor negatively significantly associated with overall lesion size or the degree of damage to LIFG. Instead, we observed a trend in which LH stroke survivors with less damage to LIFG exhibited a stronger association between more CUs/second and stronger left frontotemporal connectivity. Contrary to our predictions, the relationship between CUs/second and left intra-hemispheric connectivity was significantly stronger in individuals within the LHD group compared to the RHD group. The advantageous reliance on LH connections and non-beneficial recruitment of RH connections at a group level may reflect the fact that several people in the LHD group had relatively small lesions with minimal LIFG damage, thereby being able to rely on left intra-hemispheric connections involving LIFG for faster production of CUs. As such, it may be that RIFG activity serves a more complimentary role in language processing in some stroke survivors (66, 83), and the nature of that role as beneficial or maladaptive depends on a variety of factors, including—but likely not limited to—task type, the location and size of lesions of the participants, and the specific part of RIFG under investigation (82).

### 4.3 The role of domain-general networks in picture description informativeness and language processing

Another central finding from this study is that significant relationships between picture description measures and resting state connectivity were specific to the LN and not found for the other, domain-general networks or between LN and other network ROIs. A prevalent theory in aphasiology is that the engagement of domain-general brain regions outside the LN may promote better recovery of language in PWA (65, 66, 132), a theory supported by evidence of co-occurring engagement of domain-general and LN regions by PWA in prior activation and connectivity studies (58, 65, 83, 87, 88, 100, 111, 133–136). One proposed mechanism of action within this theory is that domain-general regions that were non-active for language processing prior to stroke assume the functions of damaged LN areas after stroke, through a process termed neurocomputational invasion (65). However, in recent meta-analyses, Wilson and Schneck (66) found little evidence supporting this theory across studies, and Stefaniak et al. (65) discovered that PWA demonstrated less activity in domain-general regions (e.g., medial superior frontal cortex, paracingulate cortex) compared to controls across language tasks, findings that undermine a neurocomputational invasion explanation of domain-general activity.

Alternatively, domain-general cognitive control networks may come online to exert top-down control of LN regions when language processing is challenging. For example, Brownsett et al. (133) found that PWA and neurologically healthy controls activated the dorsal anterior cingulate cortex and medial superior frontal gyrus to a similar extent when PWA listened to intact speech and healthy controls listened to noise-vocoded, degraded speech. In this study, greater dorsal anterior cingulate cortex activity in PWA related to better language performance. Similarly, Sharp et al. (135) found that PWA and controls exhibited similar degrees of functional connectivity between the left superior frontal gyrus and LAG when PWA listened to intact speech and healthy controls listened to degraded speech. In such instances, it is believed that domain-general regions play a supportive role in goal-directed activity when needed rather than performing linguistic operations, *per se*. Along this vein, in a series of carefully done studies using functional localizers, Fedorenko et al. (137) have found that the multiple demand network (which encompasses portions of many domain-general networks) is not engaged by neurologically healthy adults and stroke survivors without aphasia when language demands are low and specifically linguistic in nature (137–139). As such, the lack of significant associations between the CU measures and DAN, SN, DMN, and between-network connections in our study may reflect an ease with which most participants in the LHD and RHD groups completed the picture description task.

One caveat to this conclusion is that we did not employ functional localizers in the current study, and as such, the assignment of ROIs to the different networks was based on available network maps rather than subject-specific specification of language vs. domain-general regions. This is particularly important when considering the findings pertaining to LIFG given that voxels associated with linguistic vs. domain-general processes lie side by side in the frontal and parietal lobes (140, 141). Furthermore, we investigated network connectivity at the connection level, and it could be that our findings would have changed if we had considered each network as a single unit rather than comprising multiple individual connections. For example, using such an approach, Geranmayeh et al. (58) found that greater differential activity of the DMN vs. left fronto-temporo-parietal LN was associated with more appropriate information-carrying-words during a speech production task. Duncan and Small (30, 31) similarly took a network-level approach in their investigations of the relationships between treatment-induced increases in correct information units and modularity and segregation of multiple intrinsic networks. Our approach did not allow us to determine if such a network tradeoff was present in our data. These and other methodological limitations (described in “Study limitations”) are important factors to consider when designing future studies.

### 4.4 Study limitations

As referenced previously, our study did not include a comprehensive cognitive assessment administered to all participants, mainly because the study sample was gleaned retrospectively from three ongoing, prospective projects, and the testing batteries of these projects differed over the years. Relatedly, we did not have a single measure to capture motor speech impairments that if present, can affect discourse production in some stroke survivors. Nonetheless, based on the picture description recordings and the various motor speech tests included in our batteries over the years, none of the participants had pronounced dysarthria or apraxia of speech that impeded their ability to produce picture description samples. In Table 1, we reported the initial National Institutes of Health Stroke Scale [NIHSS; (142)] scores, which provide a proxy of the participants' overall stroke severity in the acute stroke phase. However, readers should apply caution in using these scores to extrapolate language deficit severity at the time of study given that the NIHSS is driven more by motor, rather than language/cognitive deficits. Furthermore, initial severity may or may not correlate with language severity at the time of study inclusion, given our participants were many months post stroke when the data for this study were collected.

Some limitations of our discourse production task and measures must also be acknowledged. There is some evidence that other types of discourse tasks (e.g., storytelling) elicit greater diversity and quantity of content compared to picture description in both PWA and neurologically healthy individuals (26, 143–145). One recommendation is to use a variety of standardized discourse tasks and measures to obtain a clear picture of an individual's discourse production abilities across genres (146, 147). However, discourse analysis can be time intensive, which is a major barrier for its implementation in routine clinical practice. In contrast, the Cookie Theft picture description task is often used in speech-language pathology and neurology as it is part of commonly-used tests in both fields [i.e., the Boston Diagnostic Aphasia Examination; Goodglass et al. (25) and the NIHSS; Brott et al. (142)] and can be easily and quickly administered without extensive specialized training. A general advantage of picture description over some discourse genres is that conceptual targets are known, and in the case of the Cookie Theft task, CUs have been predefined and extensively studied. Despite these facts, our inter-rater reliability for the percentage of interpretative CUs was only moderate compared to the excellent inter-rater reliability for the total number of CUs and CUs/second. Notably, the lower reliability for the percentage of interpretive CUs was the result of how the two raters classified synonyms of a couple of related concepts (e.g., “taking” vs. “reaching for” cookies) which in some cases, changed the numerator for some individuals and led to the discrepancy between raters. Given these collective limitations, we recommend replication of our study aims with other discourse tasks and metrics.

Another weakness of our study design was the lack of a neurologically healthy control group. While we were able to classify stroke participants as impaired or unimpaired on picture description measures based on prior normative data for Aim 1, comparing connectivity findings in the two stroke groups to a cohort of healthy individuals for Aim 2 would have provided deeper context for the impact of stroke on connectivity patterns within our participants. For example, we would expect that left intra-hemispheric connectivity would be reduced in individuals within the LHD group compared to not only people with RHD but also compared to controls, and that the same would be true of right intra-hemispheric connections for the RHD group. Given that we found no between-group differences in intrinsic network connectivity (excluding certain left LN connections) and have no control sample comparison, we cannot conclude for certain whether at a group level, these connections were initially damaged and then recovered to normal levels in both LHD and RHD groups or were hypo- or hyperconnected in both groups. Furthermore, while the functional consequences of stroke are likely most pronounced in the ipsilesional hemisphere, the lesion can have long-distance impacts on function that can result in hypoconnectivity of the contralesional hemisphere in some individuals [see e.g., (148)]. Thus, while we were still able to successfully address Aim 2 subgoals, the inclusion of a control sample could have strengthened the interpretation of our results.

## 5 Conclusions

Despite these limitations, this investigation adds to prior behavioral studies of picture description skills in stroke survivors and provides preliminary evidence for differences regarding the impact of LH vs. RH damage on relationships between intrinsic network connectivity and picture description informativeness measures. Due to its complexity, discourse production can reveal deficits missed by other neuropsychological assessments in individuals with acquired cognitive-communication disorders (149). In the future, a clearer specification of the neural substrates of different aspects of discourse may better equip clinicians in streamlining discourse assessment, scoring, and treatment protocols.

## Data availability statement

The raw data supporting the conclusions of this article will be made available by the authors, without undue reservation.

## Ethics statement

The studies involving humans were approved by the Johns Hopkins University School of Medicine Institutional Review Board. The studies were conducted in accordance with the local legislation and institutional requirements. The participants provided their written informed consent to participate in this study.

## Author contributions

EM: Conceptualization, Data curation, Formal analysis, Investigation, Methodology, Visualization, Writing—original draft, Writing—review & editing. SS: Conceptualization, Data curation, Investigation, Methodology, Writing—review & editing. RS: Conceptualization, Investigation, Methodology, Writing—review & editing. SB: Data curation, Formal analysis, Investigation, Writing—review & editing. EG: Data curation, Formal analysis, Investigation, Writing—review & editing. JS: Formal analysis, Methodology, Writing—review & editing. CS: Data curation, Formal analysis, Methodology, Writing—review & editing. AH: Conceptualization, Funding acquisition, Investigation, Methodology, Resources, Supervision, Writing—review & editing.
