# Supplementary material for: Resting state correlates of picture description informativeness in left vs. right hemisphere chronic stroke
Source: Front Neurol. 2023 Dec 7;14:1288801. doi: 10.3389/fneur.2023.1288801 (PMC10744570; doi:10.3389/fneur.2023.1288801)
Supplement: Supplementary file 1 [file Data_Sheet_1.docx]

Supplemental Table 1. Demographic and Deficit Characteristics of All Participants

| ID | Group | Sex | Age | Handedness | Education  (in years) | Initial NIHSS Score | Total CUs | Interpretative Content (%) | CUs/  second | L/R CU Ratio |
| --- | --- | --- | --- | --- | --- | --- | --- | --- | --- | --- |
| P1 | LHD | F | 37.98 | R | 14 | 2 | 10* | 40.0 | 0.435 | 1.00 |
| P2 | LHD | M | 46.84 | R | 12 | 3 | 8* | 41.7 | 0.267* | 1.00 |
| P3 | LHD | F | 50.83 | R | 12 | 3 | 13 | 38.5 | 0.325* | 0.005 |
| P4 | LHD | F | 66.12 | R | unknown | 3 | 18 | 27.8* | 0.300 | 0.63 |
| P5 | LHD | M | 36.8 | R | 12 | 12 | 0* | 0.0* | 0.000* | 0.00 |
| P6 | RHD | M | 56.43 | R | 14 | 3 | 16 | 26.1* | 0.170* | 0.92 |
| P7 | LHD | F | 63.97 | R | 16 | 4 | 19 | 31.5* | 0.244 | 2.17 |
| P8 | RHD | F | 57.85 | R | 22 | 11 | 10 | 40.0 | 1.429 | 1.00 |
| P9 | LHD | F | 80.66 | R | 11 | 1 | 7* | 28.6* | 0.132* | 1.33 |
| P10 | LHD | F | 75.23 | R | 14 | 20 | 13 | 53.8 | 0.419 | 0.63 |
| P11 | RHD | M | 66.63 | R | 5 | 5 | 12 | 41.7 | 0.923 | 0.71 |
| P12 | RHD | M | 61.82 | R | 16 | 25 | 15 | 53.6 | 0.682 | 1.50 |
| P13 | LHD | M | 30.54 | R | 12 | 20 | 7* | 57.1 | 0.108* | 1.33 |
| P14 | LHD | M | 57.11 | R | 14 | 6 | 6* | 50.0 | 0.136* | 1.00 |
| P15 | LHD | F | 57.43 | R | 14 | 0 | 14 | 21.0* | 0.298 | 1.33 |
| P16 | RHD | M | 37.14 | R | 12 | 6 | 12 | 50.0 | 0.750 | 1.40 |
| P17 | LHD | F | 71.14 | R | 12 | 6 | 7* | 57.1 | 0.108* | 1.33 |
| P18 | RHD | F | 71.1 | R | 12 | 2 | 18 | 28.0* | 0.243 | 0.80 |
| P19 | LHD | F | 79.79 | R | 17 | 5 | 17 | 23.5* | 0.224* | 0.89 |
| P20 | LHD | M | 50.98 | R | 14 | 2 | 17 | 23.5* | 0.274* | 0.89 |
| P21 | LHD | M | 69.27 | R | 18 | 5 | 20 | 0.4* | 0.213* | 0.82 |
| P22 | LHD | F | 33.13 | R | 12 | 2 | 5* | 0.0* | 0.075* | 0.67 |
| P23 | LHD | M | 58.46 | R | 14 | 7 | 17 | 29.4* | 0.362 | 0.89 |
| P24 | RHD | M | 61.46 | R | 15 | 0 | 13 | 46.2 | 0.684 | 0.64 |
| P25 | RHD | M | 65.98 | L | 14 | 4 | 12 | 41.7 | 0.273 | 1.40 |
| P26 | RHD | M | 57.38 | L | 12 | 2 | 13 | 38.5 | 1.182 | 1.60 |
| P27 | LHD | F | 55.92 | R | 15 | 3 | 14 | 57.0 | 0.378 | 0.75 |
| P28 | LHD | M | 48.83 | R | 16 | 3 | 6* | 17.0* | 0.194* | 0.20 |
| P29 | LHD | M | 53.58 | R | 18 | 2 | 15 | 40.0 | 0.500 | 0.88 |
| P30 | RHD | M | 65.32 | R | 18 | 0 | 22 | 27.3* | 0.373 | 0.83 |
| P31 | LHD | M | 56.6 | R | 16 | 5 | 13 | 30.0* | 0.108* | 1.40 |
| P32 | RHD | M | 69.53 | L | 13 | 1 | 25 | 32.0 | 0.342 | 0.79 |
| P33 | RHD | M | 76.57 | R | 15 | 2 | 3* | 67.0 | 0.200* | 0.50 |
| P34 | LHD | F | 57.55 | L | 18 | 5 | 13 | 38.5 | 0.250 | 1.17 |
| P35 | RHD | F | 25.83 | R | 16 | 1 | 15 | 41.7 | 0.682 | 1.14 |
| P36 | LHD | F | 42.33 | R | 18 | 2 | 15 | 46.7 | 0.517 | 2.00 |
| P37 | RHD | M | 59.41 | R | 22 | 0 | 24 | 25.0* | 1.000 | 1.00 |
| P38 | LHD | M | 47.58 | R | 15 | 0 | 14 | 21.4* | 0.778 | 1.33 |
| P39 | LHD | F | 62.24 | R | 9 | 9 | 9* | 44.0 | 0.900 | 0.80 |
| P40 | LHD | F | 86.93 | R | 12 | 8 | 23 | 26.0* | 0.288 | 1.08 |
| P41 | RHD | F | 51.57 | R | 10 | 9 | 15 | 13.3* | 0.833 | 1.50 |
| P42 | LHD | M | 55.8 | R | 12 | 0 | 9* | 44.0 | 0.500 | 0.80 |
| P43 | RHD | M | 69.3 | R | 12 | 7 | 9* | 22.2* | 0.818 | 2.00 |
| P44 | RHD | M | 57.56 | R | 16 | 5 | 9* | 33.3 | 0.643 | 0.50 |
| P45 | LHD | M | 60.64 | R | 12 | 5 | 17 | 13.9* | 0.152* | 2.40 |

Notes: Participants were considered impaired on a given content unit (CU) measure if they scored less than 1.5 standard deviations below the normative sample mean, as indicated by an asterisk next to the score. Separate cutoffs were calculated for younger (< 57 years old) versus older (≥ 57 years old) participants for total CUs and CUs/second based on data from Yorkston & Beukelman (1980). Prior to determining the cutoff for CUs/second, the value was converted to CUs/minute to align with the normative sample measure. The cutoff for percentage of interpretive content was derived from Myers (1979). F = female, M = male, NIHSS = National Institutes of Health Stroke Scale, L = left, R = right, L/R CU ratio = left-sided to right-sided CU ratio.

Supplemental Table 2. Stroke Characteristics of All Participants

| ID | Group | Time Post Stroke (in months) | Laterality Index | Lesion Volume (in mm^3^) | Number of Strokes | Vascular Territory | Cortical/Subcortical |
| --- | --- | --- | --- | --- | --- | --- | --- |
| P1 | LHD | 12 | 1.00 | 1688 | Single | Lenticulostriate arteries of the anterior choroidal artery | Subcortical |
| P2 | LHD | 5 | 0.98 | 8857 | Multiple | PCA, posterior communicating artery | Subcortical |
| P3 | LHD | 14 | 0.98 | 2261 | Multiple | Lenticulostriate arteries of the anterior choroidal artery | Subcortical |
| P4 | LHD | 13 | 1.00 | 2865 | Single | ACA | Cortical |
| P5 | LHD | 6 | 1.00 | 165065 | Single | MCA | Cortical |
| P6 | RHD | 13 | -1.00 | 173 | Single | Lenticulostriate arteries of the ACA, MCA, ICA and anterior choroidal artery | Subcortical |
| P7 | LHD | 5 | 1.00 | 2769 | Single | MCA | Cortical |
| P8 | RHD | 6 | -1.00 | 89297 | Single | MCA | Cortical |
| P9 | LHD | 6 | 0.90 | 3439 | Multiple | MCA | Subcortical |
| P10 | LHD | 7 | 1.00 | 641 | Single | ACA, MCA | Cortical |
| P11 | RHD | 13 | -1.00 | 2327 | Single | MCA | Cortical |
| P12 | RHD | 33 | -1.00 | 199628 | Single | MCA | Cortical |
| P13 | LHD | 8 | 1.00 | 36049 | Single | MCA | Cortical |
| P14 | LHD | 12 | 1.00 | 95348 | Single | MCA | Cortical |
| P15 | LHD | 13 | 1.00 | 933 | Single | PCA | Cortical |
| P16 | RHD | 6 | -1.00 | 64684 | Single | MCA | Cortical |
| P17 | LHD | 11 | 0.99 | 15125 | Single | MCA | Cortical |
| P18 | RHD | 11 | -0.79 | 1749 | Multiple | Lenticulostriate arteries of the ACA, MCA, ICA and anterior choroidal artery | Subcortical |
| P19 | LHD | 6 | 0.82 | 274 | Single | MCA | Cortical |
| P20 | LHD | 12 | 0.95 | 19762 | Single | PCA | Cortical |
| P21 | LHD | 7 | 1.00 | 92 | Single | MCA | Cortical |
| P22 | LHD | 5 | 0.99 | 145761 | Single | MCA | Cortical |
| P23 | LHD | 14 | 1.00 | 22734 | Single | MCA | Cortical |
| P24 | RHD | 15 | -1.00 | 139 | Single | PCA, posterior communicating artery | Subcortical |
| P25 | RHD | 12 | -0.84 | 508 | Multiple | Lenticulostriate arteries of the ACA, MCA, ICA and anterior choroidal artery | Subcortical |
| P26 | RHD | 4 | -1.00 | 104 | Single | Lenticulostriate arteries of the anterior choroidal and insular arteries | Subcortical |
| P27 | LHD | 11 | 0.98 | 1306 | Single | PCA | Cortical |
| P28 | LHD | 13 | 0.99 | 40324 | Single | PCA | Cortical |
| P29 | LHD | 13 | 1.00 | 34 | Single | PCA | Subcortical |
| P30 | RHD | 15 | -1.00 | 2571 | Single | PCA | Cortical |
| P31 | LHD | 14 | 1.00 | 164691 | Single | MCA | Cortical |
| P32 | RHD | 13 | -1.00 | 10770 | Single | PCA | Cortical |
| P33 | RHD | 6 | -1.00 | 23247 | Single | MCA | Cortical |
| P34 | LHD | 14 | 1.00 | 1325 | Single | MCA | Subcortical |
| P35 | RHD | 14 | -1.00 | 314 | Single | MCA | Cortical |
| P36 | LHD | 13 | 1.00 | 13962 | Single | MCA | Cortical |
| P37 | RHD | 7 | -1.00 | 80 | Single | MCA | Cortical |
| P38 | LHD | 5 | 1.00 | 3330 | Single | Vertebrobasilar artery circulation (multifocal infarct) | Cortical |
| P39 | LHD | 14 | 1.00 | 15948 | Single | ACA, MCA | Cortical |
| P40 | LHD | 13 | 1.00 | 1576 | Single | MCA | Cortical |
| P41 | RHD | 4 | -1.00 | 56220 | Single | MCA | Cortical |
| P42 | LHD | 13 | 0.98 | 466 | Single | Lenticulostriate arteries of the ACA, MCA, ICA and anterior choroidal artery | Subcortical |
| P43 | RHD | 15 | -1.00 | 77154 | Single | MCA | Cortical |
| P44 | RHD | 12 | -1.00 | 5771 | Single | MCA | Cortical |
| P45 | LHD | 6 | 1.00 | 117 | Single | PCA, posterior communicating artery | Subcortical |

Notes: A laterality index of -1.00 is indicative of right hemisphere damage (RHD) and no left hemisphere damage (LHD) whereas a laterality index of +1.00 indicates the opposite. ACA = anterior cerebral artery, ICA = internal carotid artery, MCA = middle cerebral artery, PCA = posterior cerebral artery.

Supplemental Table 3. Percent Damage to the Dorsal Attention Network Regions

| ID | Group | LFEF | LIPS | RFEF | RIPS | Total DAN Damage (%) |
| --- | --- | --- | --- | --- | --- | --- |
| P1 | LHD | 0.00 | 0.00 | 0.00 | 0.00 | 0.00 |
| P2 | LHD | 0.00 | 0.00 | 0.00 | 0.00 | 0.00 |
| P3 | LHD | 0.00 | 0.00 | 0.00 | 0.00 | 0.00 |
| P4 | LHD | 0.00 | 0.00 | 0.00 | 0.00 | 0.00 |
| P5 | LHD | 0.00 | 39.17 | 0.00 | 0.00 | 19.77 |
| P6 | RHD | 0.00 | 0.00 | 0.00 | 0.00 | 0.00 |
| P7 | LHD | 0.00 | 0.00 | 0.00 | 0.00 | 0.00 |
| P8 | RHD | 0.00 | 0.00 | 0.00 | 0.00 | 0.00 |
| P9 | LHD | 0.00 | 0.00 | 0.00 | 0.00 | 0.00 |
| P10 | LHD | 0.00 | 0.00 | 0.00 | 0.00 | 0.00 |
| P11 | RHD | 0.00 | 0.00 | 0.00 | 0.00 | 0.00 |
| P12 | RHD | 0.00 | 0.00 | 0.00 | 13.75 | 6.51 |
| P13 | LHD | 0.00 | 0.14 | 0.00 | 0.00 | 0.07 |
| P14 | LHD | 6.63 | 1.08 | 0.00 | 0.00 | 0.64 |
| P15 | LHD | 0.00 | 0.00 | 0.00 | 0.00 | 0.00 |
| P16 | RHD | 0.00 | 0.00 | 0.00 | 0.00 | 0.00 |
| P17 | LHD | 0.00 | 6.57 | 0.00 | 0.00 | 3.31 |
| P18 | RHD | 0.00 | 0.00 | 0.00 | 0.00 | 0.00 |
| P19 | LHD | 0.00 | 0.00 | 0.00 | 0.00 | 0.00 |
| P20 | LHD | 0.00 | 0.00 | 0.00 | 0.00 | 0.00 |
| P21 | LHD | 0.00 | 0.00 | 0.00 | 0.00 | 0.00 |
| P22 | LHD | 0.00 | 8.65 | 0.00 | 0.00 | 4.37 |
| P23 | LHD | 0.00 | 3.61 | 0.00 | 0.00 | 1.82 |
| P24 | RHD | 0.00 | 0.00 | 0.00 | 0.00 | 0.00 |
| P25 | RHD | 0.00 | 0.00 | 0.00 | 0.00 | 0.00 |
| P26 | RHD | 0.00 | 0.00 | 0.00 | 0.00 | 0.00 |
| P27 | LHD | 0.00 | 0.00 | 0.00 | 0.00 | 0.00 |
| P28 | LHD | 0.00 | 0.18 | 0.00 | 0.00 | 0.09 |
| P29 | LHD | 0.00 | 0.00 | 0.00 | 0.00 | 0.00 |
| P30 | RHD | 0.00 | 0.00 | 0.00 | 0.00 | 0.00 |
| P31 | LHD | 0.00 | 72.50 | 0.00 | 0.00 | 36.59 |
| P32 | RHD | 0.00 | 0.00 | 0.00 | 0.00 | 0.00 |
| P33 | RHD | 0.00 | 0.00 | 0.00 | 0.00 | 0.00 |
| P34 | LHD | 0.00 | 0.00 | 0.00 | 0.00 | 0.00 |
| P35 | RHD | 0.00 | 0.00 | 0.00 | 0.00 | 0.00 |
| P36 | LHD | 0.00 | 6.18 | 0.00 | 0.00 | 3.12 |
| P37 | RHD | 0.00 | 0.00 | 0.00 | 0.34 | 0.16 |
| P38 | LHD | 0.00 | 0.00 | 0.00 | 0.00 | 0.00 |
| P39 | LHD | 0.00 | 0.00 | 0.00 | 0.00 | 0.00 |
| P40 | LHD | 0.00 | 0.00 | 0.00 | 0.00 | 0.00 |
| P41 | RHD | 0.00 | 0.00 | 0.00 | 30.53 | 14.46 |
| P42 | LHD | 0.00 | 0.00 | 0.00 | 0.00 | 0.00 |
| P43 | RHD | 0.00 | 0.00 | 31.60 | 10.16 | 5.07 |
| P44 | RHD | 0.00 | 0.00 | 0.00 | 0.00 | 0.00 |
| P45 | LHD | 0.00 | 0.00 | 0.00 | 0.00 | 0.00 |

Notes: LHD = left hemisphere damage, RHD = right hemisphere damage, L = left, right = right, FEF = frontal eye field, IPS = intraparietal sulcus, DAN = dorsal attention network

Supplemental Table 4. Percent Damage to the Default Mode Network Regions

| ID | Group | LLP | MPFC | PCC | RLP | Total DMN Damage (%) |
| --- | --- | --- | --- | --- | --- | --- |
| P1 | LHD | 0.00 | 0.00 | 0.00 | 0.00 | 0.00 |
| P2 | LHD | 0.00 | 0.00 | 0.00 | 0.00 | 0.00 |
| P3 | LHD | 0.00 | 0.00 | 0.00 | 0.00 | 0.00 |
| P4 | LHD | 0.00 | 0.00 | 0.00 | 0.00 | 0.00 |
| P5 | LHD | 49.04 | 0.00 | 0.96 | 0.00 | 6.58 |
| P6 | RHD | 0.00 | 0.00 | 0.00 | 0.00 | 0.00 |
| P7 | LHD | 0.00 | 0.00 | 0.00 | 0.00 | 0.00 |
| P8 | RHD | 0.00 | 0.00 | 0.00 | 57.67 | 8.87 |
| P9 | LHD | 0.00 | 0.00 | 0.00 | 0.00 | 0.00 |
| P10 | LHD | 0.00 | 0.00 | 0.00 | 0.00 | 0.00 |
| P11 | RHD | 0.00 | 0.00 | 0.00 | 0.00 | 0.00 |
| P12 | RHD | 0.00 | 0.00 | 0.00 | 24.71 | 3.80 |
| P13 | LHD | 0.00 | 0.00 | 0.00 | 0.00 | 0.00 |
| P14 | LHD | 56.41 | 0.00 | 0.00 | 0.00 | 6.95 |
| P15 | LHD | 0.00 | 0.00 | 0.00 | 0.00 | 0.00 |
| P16 | RHD | 0.00 | 0.00 | 0.00 | 0.00 | 0.00 |
| P17 | LHD | 0.00 | 0.00 | 0.00 | 0.00 | 0.00 |
| P18 | RHD | 0.00 | 0.00 | 0.00 | 0.00 | 0.00 |
| P19 | LHD | 0.00 | 0.00 | 0.00 | 0.00 | 0.00 |
| P20 | LHD | 0.00 | 0.00 | 0.12 | 0.00 | 0.07 |
| P21 | LHD | 0.00 | 0.00 | 0.00 | 0.00 | 0.00 |
| P22 | LHD | 67.19 | 0.00 | 0.00 | 0.00 | 8.28 |
| P23 | LHD | 0.00 | 0.00 | 0.00 | 0.00 | 0.00 |
| P24 | RHD | 0.00 | 0.00 | 0.00 | 0.00 | 0.00 |
| P25 | RHD | 0.00 | 0.00 | 0.00 | 0.00 | 0.00 |
| P26 | RHD | 0.00 | 0.00 | 0.00 | 0.00 | 0.00 |
| P27 | LHD | 0.00 | 0.00 | 0.01 | 0.00 | 0.01 |
| P28 | LHD | 0.00 | 0.00 | 13.85 | 0.00 | 7.80 |
| P29 | LHD | 0.00 | 0.00 | 0.00 | 0.00 | 0.00 |
| P30 | RHD | 0.00 | 0.00 | 0.00 | 0.00 | 0.00 |
| P31 | LHD | 88.81 | 0.00 | 0.22 | 0.00 | 11.06 |
| P32 | RHD | 0.00 | 0.00 | 0.00 | 0.00 | 0.00 |
| P33 | RHD | 0.00 | 0.00 | 0.00 | 0.00 | 0.00 |
| P34 | LHD | 0.00 | 0.00 | 0.00 | 0.00 | 0.00 |
| P35 | RHD | 0.00 | 0.00 | 0.00 | 0.00 | 0.00 |
| P36 | LHD | 0.06 | 0.00 | 0.00 | 0.00 | 0.01 |
| P37 | RHD | 0.00 | 0.00 | 0.00 | 0.00 | 0.00 |
| P38 | LHD | 0.00 | 0.00 | 0.00 | 0.00 | 0.00 |
| P39 | LHD | 27.54 | 0.00 | 0.00 | 0.00 | 3.39 |
| P40 | LHD | 0.00 | 0.00 | 0.00 | 0.00 | 0.00 |
| P41 | RHD | 0.00 | 0.00 | 0.00 | 10.04 | 1.54 |
| P42 | LHD | 0.00 | 0.00 | 0.00 | 0.00 | 0.00 |
| P43 | RHD | 0.00 | 0.00 | 1.20 | 72.76 | 11.87 |
| P44 | RHD | 0.00 | 0.00 | 0.00 | 0.00 | 0.00 |
| P45 | LHD | 0.00 | 0.00 | 0.00 | 0.00 | 0.00 |

Notes: LHD = left hemisphere damage, RHD = right hemisphere damage, L = left, R = right, LP = lateral parietal cortex, MPFC = medial prefrontal cortex, PCC = posterior cingulate cortex, DMN = default mode network

Supplemental Table 5. Percent Damage to Left Hemisphere Language Network Regions

| ID | Group | MFG | IFGop | IFGorb | IFGtri | STpole | STG | pSTG | MTpole | MTG | pMTG | ITG | pITG | SMG | AG | Total LH Damage (%) |
| --- | --- | --- | --- | --- | --- | --- | --- | --- | --- | --- | --- | --- | --- | --- | --- | --- |
| P1 | LHD | 0.00 | 0.00 | 0.00 | 0.00 | 0.00 | 0.00 | 0.00 | 0.00 | 0.00 | 0.00 | 0.00 | 0.00 | 0.00 | 0.00 | 0.00 |
| P2 | LHD | 1.85 | 3.88 | 0.00 | 1.88 | 0.00 | 0.00 | 0.00 | 0.00 | 0.00 | 0.00 | 0.00 | 0.00 | 0.00 | 0.00 | 0.00 |
| P3 | LHD | 0.00 | 0.00 | 0.00 | 0.00 | 0.00 | 0.00 | 0.00 | 0.00 | 0.00 | 0.00 | 0.00 | 0.00 | 0.00 | 0.00 | 0.00 |
| P4 | LHD | 0.00 | 0.00 | 0.00 | 0.00 | 0.00 | 0.00 | 0.00 | 0.00 | 0.00 | 0.00 | 0.00 | 0.00 | 0.00 | 0.00 | 0.00 |
| P5 | LHD | 33.94 | 88.97 | 3.31 | 44.68 | 0.61 | 34.14 | 99.20 | 0.00 | 0.19 | 69.02 | 0.00 | 1.59 | 74.03 | 93.21 | 0.00 |
| P6 | RHD | 0.00 | 0.00 | 0.00 | 0.00 | 0.00 | 0.00 | 0.00 | 0.00 | 0.00 | 0.00 | 0.00 | 0.00 | 0.00 | 0.00 | 0.00 |
| P7 | LHD | 0.00 | 0.00 | 0.00 | 0.00 | 0.00 | 0.16 | 0.00 | 0.00 | 0.00 | 0.00 | 0.00 | 0.00 | 0.00 | 0.00 | 0.00 |
| P8 | RHD | 0.00 | 0.00 | 0.00 | 0.00 | 0.00 | 0.00 | 0.00 | 0.00 | 0.00 | 0.00 | 0.00 | 0.00 | 0.00 | 0.00 | 27.95 |
| P9 | LHD | 0.14 | 0.00 | 0.00 | 0.00 | 0.00 | 0.00 | 0.00 | 0.00 | 0.00 | 0.00 | 0.00 | 0.00 | 0.00 | 0.00 | 0.00 |
| P10 | LHD | 0.04 | 0.04 | 0.39 | 0.10 | 0.00 | 0.00 | 0.00 | 0.00 | 0.00 | 0.00 | 0.00 | 0.00 | 0.00 | 0.00 | 0.00 |
| P11 | RHD | 0.00 | 0.00 | 0.00 | 0.00 | 0.00 | 0.00 | 0.00 | 0.00 | 0.00 | 0.00 | 0.00 | 0.00 | 0.00 | 0.00 | 0.00 |
| P12 | RHD | 0.00 | 0.00 | 0.00 | 0.00 | 0.00 | 0.00 | 0.00 | 0.00 | 0.00 | 0.00 | 0.00 | 0.00 | 0.00 | 0.00 | 57.28 |
| P13 | LHD | 0.66 | 5.76 | 1.23 | 4.45 | 0.37 | 32.79 | 20.69 | 0.00 | 0.44 | 0.00 | 0.04 | 0.00 | 10.02 | 0.00 | 0.00 |
| P14 | LHD | 11.79 | 0.05 | 0.00 | 0.00 | 5.45 | 51.93 | 87.65 | 0.28 | 11.04 | 69.74 | 0.03 | 3.84 | 9.81 | 62.74 | 0.00 |
| P15 | LHD | 0.00 | 0.00 | 0.00 | 0.00 | 0.00 | 0.00 | 0.00 | 0.00 | 0.00 | 0.00 | 0.00 | 0.00 | 0.00 | 0.00 | 0.00 |
| P16 | RHD | 0.00 | 0.00 | 0.00 | 0.00 | 0.00 | 0.00 | 0.00 | 0.00 | 0.00 | 0.00 | 0.00 | 0.00 | 0.00 | 0.00 | 23.98 |
| P17 | LHD | 17.97 | 1.22 | 0.00 | 0.00 | 0.00 | 0.61 | 0.46 | 0.00 | 0.00 | 0.00 | 0.00 | 0.00 | 18.78 | 0.00 | 0.00 |
| P18 | RHD | 0.00 | 0.00 | 0.00 | 0.00 | 0.00 | 0.00 | 0.00 | 0.00 | 0.00 | 0.00 | 0.00 | 0.00 | 0.00 | 0.00 | 0.00 |
| P19 | LHD | 0.00 | 0.00 | 0.00 | 0.00 | 0.00 | 0.00 | 0.00 | 0.00 | 0.00 | 0.00 | 0.00 | 0.00 | 0.00 | 0.00 | 0.00 |
| P20 | LHD | 0.00 | 0.00 | 0.00 | 0.00 | 0.00 | 0.00 | 0.00 | 0.00 | 0.00 | 0.00 | 0.00 | 0.00 | 0.00 | 0.00 | 0.00 |
| P21 | LHD | 0.00 | 0.00 | 0.00 | 0.00 | 0.00 | 0.00 | 0.00 | 0.00 | 0.00 | 0.00 | 0.00 | 0.00 | 0.00 | 0.00 | 0.00 |
| P22 | LHD | 8.26 | 10.95 | 0.00 | 0.74 | 36.34 | 40.48 | 44.34 | 68.65 | 81.19 | 90.83 | 70.55 | 98.29 | 23.03 | 26.95 | 0.00 |
| P23 | LHD | 8.25 | 19.18 | 22.01 | 32.82 | 0.00 | 0.00 | 0.28 | 0.00 | 0.00 | 0.00 | 0.00 | 0.00 | 8.69 | 2.55 | 0.00 |
| P24 | RHD | 0.00 | 0.00 | 0.00 | 0.00 | 0.00 | 0.00 | 0.00 | 0.00 | 0.00 | 0.00 | 0.00 | 0.00 | 0.00 | 0.00 | 0.00 |
| P25 | RHD | 0.00 | 0.00 | 0.00 | 0.00 | 0.00 | 0.00 | 0.00 | 0.00 | 0.00 | 0.00 | 0.00 | 0.00 | 0.00 | 0.00 | 0.00 |
| P26 | RHD | 0.00 | 0.00 | 0.00 | 0.00 | 0.00 | 0.00 | 0.00 | 0.00 | 0.00 | 0.00 | 0.00 | 0.00 | 0.00 | 0.00 | 0.00 |
| P27 | LHD | 0.00 | 0.00 | 0.00 | 0.00 | 0.00 | 0.00 | 0.00 | 0.00 | 0.00 | 0.00 | 0.00 | 0.00 | 0.00 | 0.00 | 0.00 |
| P28 | LHD | 0.00 | 0.00 | 0.00 | 0.00 | 0.00 | 0.00 | 0.00 | 0.00 | 0.00 | 0.47 | 0.00 | 0.00 | 0.00 | 0.00 | 0.00 |
| P29 | LHD | 0.00 | 0.00 | 0.00 | 0.00 | 0.00 | 0.00 | 0.00 | 0.00 | 0.00 | 0.00 | 0.00 | 0.00 | 0.00 | 0.00 | 0.00 |
| P30 | RHD | 0.00 | 0.00 | 0.00 | 0.00 | 0.00 | 0.00 | 0.00 | 0.00 | 0.00 | 0.00 | 0.00 | 0.00 | 0.00 | 0.00 | 0.00 |
| P31 | LHD | 0.00 | 0.11 | 0.11 | 0.00 | 33.66 | 74.95 | 75.06 | 5.00 | 82.00 | 78.61 | 21.99 | 68.90 | 87.55 | 97.86 | 0.00 |
| P32 | RHD | 0.00 | 0.00 | 0.00 | 0.00 | 0.00 | 0.00 | 0.00 | 0.00 | 0.00 | 0.00 | 0.00 | 0.00 | 0.00 | 0.00 | 1.31 |
| P33 | RHD | 0.00 | 0.00 | 0.00 | 0.00 | 0.00 | 0.00 | 0.00 | 0.00 | 0.00 | 0.00 | 0.00 | 0.00 | 0.00 | 0.00 | 7.52 |
| P34 | LHD | 0.00 | 0.00 | 0.00 | 0.00 | 0.00 | 0.00 | 0.00 | 0.00 | 0.00 | 0.00 | 0.00 | 0.00 | 0.00 | 0.00 | 0.00 |
| P35 | RHD | 0.00 | 0.00 | 0.00 | 0.00 | 0.00 | 0.00 | 0.00 | 0.00 | 0.00 | 0.00 | 0.00 | 0.00 | 0.00 | 0.00 | 0.00 |
| P36 | LHD | 4.25 | 6.84 | 0.00 | 0.00 | 0.00 | 0.00 | 0.00 | 0.00 | 0.00 | 3.18 | 0.00 | 0.00 | 7.42 | 0.00 | 0.00 |
| P37 | RHD | 0.00 | 0.00 | 0.00 | 0.00 | 0.00 | 0.00 | 0.00 | 0.00 | 0.00 | 0.00 | 0.00 | 0.00 | 0.00 | 0.00 | 0.04 |
| P38 | LHD | 0.10 | 0.00 | 0.00 | 0.00 | 0.00 | 0.00 | 0.00 | 0.00 | 0.00 | 0.00 | 0.00 | 0.00 | 0.00 | 0.00 | 0.00 |
| P39 | LHD | 0.12 | 0.00 | 0.00 | 0.00 | 0.00 | 0.00 | 0.00 | 0.00 | 0.00 | 4.84 | 0.00 | 0.00 | 0.00 | 0.04 | 0.00 |
| P40 | LHD | 0.00 | 0.00 | 0.00 | 0.00 | 0.00 | 0.00 | 0.00 | 0.00 | 0.00 | 0.00 | 0.00 | 0.00 | 0.00 | 0.00 | 0.00 |
| P41 | RHD | 0.00 | 0.00 | 0.00 | 0.00 | 0.00 | 0.00 | 0.00 | 0.00 | 0.00 | 0.00 | 0.00 | 0.00 | 0.00 | 0.00 | 18.47 |
| P42 | LHD | 0.00 | 0.00 | 0.00 | 0.00 | 0.00 | 0.00 | 0.00 | 0.00 | 0.00 | 0.00 | 0.00 | 0.00 | 0.00 | 0.00 | 0.00 |
| P43 | RHD | 0.00 | 0.00 | 0.00 | 0.00 | 0.00 | 0.00 | 0.00 | 0.00 | 0.00 | 0.00 | 0.00 | 0.00 | 0.00 | 0.00 | 17.67 |
| P44 | RHD | 0.00 | 0.00 | 0.00 | 0.00 | 0.00 | 0.00 | 0.00 | 0.00 | 0.00 | 0.00 | 0.00 | 0.00 | 0.00 | 0.00 | 0.00 |
| P45 | LHD | 0.00 | 0.00 | 0.00 | 0.00 | 0.00 | 0.00 | 0.00 | 0.00 | 0.00 | 0.00 | 0.00 | 0.00 | 0.00 | 0.00 | 0.00 |

Notes: LHD = left hemisphere damage, RHD = right hemisphere damage, MFG = middle frontal gyrus, IFGop = inferior frontal gyrus, pars opercularis, IFGorb = IFG, pars orbitalis, IFGtri = IFG, pars triangularis, STpole = superior temporal pole, STG = superior temporal gyrus, p = posterior, MTpole = middle temporal pole, MTG = middle temporal gyrus, ITG = inferior temporal gyrus, SMG = supramarginal gyrus, AG = angular gyrus, LN = language network

Supplemental Table 6. Percent Damage to Right Hemisphere Language Network Regions

| ID | Group | MFG | IFGop | IFGorb | IFGtri | STpole | STG | pSTG | MTpole | MTG | pMTG | ITG | pITG | SMG | AG | Total RH Damage (%) |
| --- | --- | --- | --- | --- | --- | --- | --- | --- | --- | --- | --- | --- | --- | --- | --- | --- |
| P1 | LHD | 0.00 | 0.00 | 0.00 | 0.00 | 0.00 | 0.00 | 0.00 | 0.00 | 0.00 | 0.00 | 0.00 | 0.00 | 0.00 | 0.00 | 0.00 |
| P2 | LHD | 0.00 | 0.00 | 0.00 | 0.00 | 0.00 | 0.00 | 0.00 | 0.00 | 0.00 | 0.00 | 0.00 | 0.00 | 0.00 | 0.00 | 0.00 |
| P3 | LHD | 0.00 | 0.00 | 0.00 | 0.00 | 0.00 | 0.00 | 0.00 | 0.00 | 0.00 | 0.00 | 0.00 | 0.00 | 0.00 | 0.00 | 0.00 |
| P4 | LHD | 0.00 | 0.00 | 0.00 | 0.00 | 0.00 | 0.00 | 0.00 | 0.00 | 0.00 | 0.00 | 0.00 | 0.00 | 0.00 | 0.00 | 0.00 |
| P5 | LHD | 0.00 | 0.00 | 0.00 | 0.00 | 0.00 | 0.00 | 0.00 | 0.00 | 0.00 | 0.00 | 0.00 | 0.00 | 0.00 | 0.00 | 0.00 |
| P6 | RHD | 0.00 | 0.00 | 0.00 | 0.00 | 0.00 | 0.00 | 0.00 | 0.00 | 0.00 | 0.00 | 0.00 | 0.00 | 0.00 | 0.00 | 0.00 |
| P7 | LHD | 0.00 | 0.00 | 0.00 | 0.00 | 0.00 | 0.00 | 0.00 | 0.00 | 0.00 | 0.00 | 0.00 | 0.00 | 0.00 | 0.00 | 0.00 |
| P8 | RHD | 0.00 | 20.49 | 2.52 | 0.93 | 45.18 | 85.13 | 65.53 | 2.50 | 46.95 | 63.65 | 0.04 | 0.62 | 23.02 | 25.68 | 27.95 |
| P9 | LHD | 0.00 | 0.00 | 0.00 | 0.00 | 0.00 | 0.00 | 0.00 | 0.00 | 0.00 | 0.00 | 0.00 | 0.00 | 0.00 | 0.00 | 0.00 |
| P10 | LHD | 0.00 | 0.00 | 0.00 | 0.00 | 0.00 | 0.00 | 0.00 | 0.00 | 0.00 | 0.00 | 0.00 | 0.00 | 0.00 | 0.00 | 0.00 |
| P11 | RHD | 0.00 | 0.00 | 0.00 | 0.00 | 0.00 | 0.00 | 0.00 | 0.00 | 0.00 | 0.00 | 0.00 | 0.00 | 0.00 | 0.00 | 0.00 |
| P12 | RHD | 13.24 | 95.74 | 98.34 | 96.56 | 95.92 | 94.44 | 76.19 | 81.31 | 67.59 | 46.09 | 9.07 | 0.26 | 80.99 | 42.05 | 57.28 |
| P13 | LHD | 0.00 | 0.00 | 0.00 | 0.00 | 0.00 | 0.00 | 0.00 | 0.00 | 0.00 | 0.00 | 0.00 | 0.00 | 0.00 | 0.00 | 0.00 |
| P14 | LHD | 0.00 | 0.00 | 0.00 | 0.00 | 0.00 | 0.00 | 0.00 | 0.00 | 0.00 | 0.00 | 0.00 | 0.00 | 0.00 | 0.00 | 0.00 |
| P15 | LHD | 0.00 | 0.00 | 0.00 | 0.00 | 0.00 | 0.00 | 0.00 | 0.00 | 0.00 | 0.00 | 0.00 | 0.00 | 0.00 | 0.00 | 0.00 |
| P16 | RHD | 51.88 | 86.89 | 3.00 | 80.63 | 87.72 | 31.19 | 0.02 | 52.60 | 0.01 | 0.00 | 2.75 | 0.00 | 0.00 | 0.00 | 23.98 |
| P17 | LHD | 0.00 | 0.00 | 0.00 | 0.00 | 0.00 | 0.00 | 0.00 | 0.00 | 0.00 | 0.00 | 0.00 | 0.00 | 0.00 | 0.00 | 0.00 |
| P18 | RHD | 0.00 | 0.00 | 0.00 | 0.00 | 0.00 | 0.00 | 0.00 | 0.00 | 0.00 | 0.00 | 0.02 | 0.00 | 0.00 | 0.00 | 0.00 |
| P19 | LHD | 0.00 | 0.00 | 0.00 | 0.00 | 0.00 | 0.00 | 0.00 | 0.00 | 0.00 | 0.00 | 0.00 | 0.00 | 0.00 | 0.00 | 0.00 |
| P20 | LHD | 0.00 | 0.00 | 0.00 | 0.00 | 0.00 | 0.00 | 0.00 | 0.00 | 0.00 | 0.00 | 0.00 | 0.00 | 0.00 | 0.00 | 0.00 |
| P21 | LHD | 0.00 | 0.00 | 0.00 | 0.00 | 0.00 | 0.00 | 0.00 | 0.00 | 0.00 | 0.00 | 0.00 | 0.00 | 0.00 | 0.00 | 0.00 |
| P22 | LHD | 0.00 | 0.00 | 0.00 | 0.00 | 0.00 | 0.00 | 0.00 | 0.00 | 0.00 | 0.00 | 0.00 | 0.00 | 0.00 | 0.00 | 0.00 |
| P23 | LHD | 0.00 | 0.00 | 0.00 | 0.00 | 0.00 | 0.00 | 0.00 | 0.00 | 0.00 | 0.00 | 0.00 | 0.00 | 0.00 | 0.00 | 0.00 |
| P24 | RHD | 0.00 | 0.00 | 0.00 | 0.00 | 0.00 | 0.00 | 0.00 | 0.00 | 0.00 | 0.00 | 0.00 | 0.00 | 0.00 | 0.00 | 0.00 |
| P25 | RHD | 0.00 | 0.00 | 0.00 | 0.00 | 0.00 | 0.00 | 0.00 | 0.00 | 0.00 | 0.00 | 0.00 | 0.00 | 0.00 | 0.00 | 0.00 |
| P26 | RHD | 0.00 | 0.00 | 0.00 | 0.00 | 0.00 | 0.00 | 0.00 | 0.00 | 0.00 | 0.00 | 0.00 | 0.00 | 0.00 | 0.00 | 0.00 |
| P27 | LHD | 0.00 | 0.00 | 0.00 | 0.00 | 0.00 | 0.00 | 0.00 | 0.00 | 0.00 | 0.00 | 0.00 | 0.00 | 0.00 | 0.00 | 0.00 |
| P28 | LHD | 0.00 | 0.00 | 0.00 | 0.00 | 0.00 | 0.00 | 0.00 | 0.00 | 0.00 | 0.00 | 0.00 | 0.00 | 0.00 | 0.00 | 0.00 |
| P29 | LHD | 0.00 | 0.00 | 0.00 | 0.00 | 0.00 | 0.00 | 0.00 | 0.00 | 0.00 | 0.00 | 0.00 | 0.00 | 0.00 | 0.00 | 0.00 |
| P30 | RHD | 0.00 | 0.00 | 0.00 | 0.00 | 0.00 | 0.00 | 0.00 | 0.00 | 0.00 | 0.00 | 0.00 | 0.00 | 0.00 | 0.00 | 0.00 |
| P31 | LHD | 0.00 | 0.00 | 0.00 | 0.00 | 0.00 | 0.00 | 0.00 | 0.00 | 0.00 | 0.00 | 0.00 | 0.00 | 0.00 | 0.00 | 0.00 |
| P32 | RHD | 0.00 | 0.00 | 0.00 | 0.00 | 0.00 | 0.00 | 0.00 | 0.00 | 0.00 | 0.00 | 0.00 | 24.70 | 0.00 | 0.00 | 1.31 |
| P33 | RHD | 0.00 | 0.03 | 0.00 | 0.16 | 7.12 | 25.85 | 11.13 | 0.43 | 62.54 | 14.53 | 1.47 | 1.03 | 0.00 | 0.00 | 7.52 |
| P34 | LHD | 0.00 | 0.00 | 0.00 | 0.00 | 0.00 | 0.00 | 0.00 | 0.00 | 0.00 | 0.00 | 0.00 | 0.00 | 0.00 | 0.00 | 0.00 |
| P35 | RHD | 0.00 | 0.00 | 0.00 | 0.00 | 0.00 | 0.00 | 0.00 | 0.00 | 0.00 | 0.00 | 0.00 | 0.00 | 0.00 | 0.00 | 0.00 |
| P36 | LHD | 0.00 | 0.00 | 0.00 | 0.00 | 0.00 | 0.00 | 0.00 | 0.00 | 0.00 | 0.00 | 0.00 | 0.00 | 0.00 | 0.00 | 0.00 |
| P37 | RHD | 0.00 | 0.00 | 0.00 | 0.00 | 0.00 | 0.00 | 0.00 | 0.00 | 0.00 | 0.00 | 0.00 | 0.00 | 0.39 | 0.00 | 0.04 |
| P38 | LHD | 0.00 | 0.00 | 0.00 | 0.00 | 0.00 | 0.00 | 0.00 | 0.00 | 0.00 | 0.00 | 0.00 | 0.00 | 0.00 | 0.00 | 0.00 |
| P39 | LHD | 0.00 | 0.00 | 0.00 | 0.00 | 0.00 | 0.00 | 0.00 | 0.00 | 0.00 | 0.00 | 0.00 | 0.00 | 0.00 | 0.00 | 0.00 |
| P40 | LHD | 0.00 | 0.00 | 0.00 | 0.00 | 0.00 | 0.00 | 0.00 | 0.00 | 0.00 | 0.00 | 0.00 | 0.00 | 0.00 | 0.00 | 0.00 |
| P41 | RHD | 13.39 | 49.53 | 0.00 | 3.28 | 0.00 | 0.28 | 10.49 | 0.00 | 0.00 | 0.12 | 0.00 | 0.00 | 66.31 | 42.66 | 18.47 |
| P42 | LHD | 0.00 | 0.00 | 0.00 | 0.00 | 0.00 | 0.00 | 0.00 | 0.00 | 0.00 | 0.00 | 0.00 | 0.00 | 0.00 | 0.00 | 0.00 |
| P43 | RHD | 0.01 | 0.00 | 0.00 | 0.00 | 0.00 | 3.50 | 39.29 | 0.00 | 0.00 | 23.39 | 0.00 | 0.00 | 14.91 | 74.11 | 17.67 |
| P44 | RHD | 0.00 | 0.00 | 0.00 | 0.00 | 0.00 | 0.00 | 0.00 | 0.00 | 0.00 | 0.00 | 0.00 | 0.00 | 0.00 | 0.00 | 0.00 |
| P45 | LHD | 0.00 | 0.00 | 0.00 | 0.00 | 0.00 | 0.00 | 0.00 | 0.00 | 0.00 | 0.00 | 0.00 | 0.00 | 0.00 | 0.00 | 0.00 |

Notes: LHD = left hemisphere damage, RHD = right hemisphere damage, MFG = middle frontal gyrus, IFGop = inferior frontal gyrus, pars opercularis, IFGorb = IFG, pars orbitalis, IFGtri = IFG, pars triangularis, STpole = superior temporal pole, STG = superior temporal gyrus, p = posterior, MTpole = middle temporal pole, MTG = middle temporal gyrus, ITG = inferior temporal gyrus, SMG = supramarginal gyrus, AG = angular gyrus, LN = language network

Supplemental Table 7. Percent Damage to Salience Network Regions

| ID | Group | ACC | LRPFC | LaInsula | LSMG | RRPFC | RaInsula | RSMG | Total Damage (%) |
| --- | --- | --- | --- | --- | --- | --- | --- | --- | --- |
| P1 | LHD | 0.00 | 0.00 | 0.00 | 0.00 | 0.00 | 0.00 | 0.00 | 0.00 |
| P2 | LHD | 0.00 | 0.00 | 0.00 | 0.00 | 0.00 | 0.00 | 0.00 | 0.00 |
| P3 | LHD | 0.00 | 0.00 | 0.00 | 0.00 | 0.00 | 0.00 | 0.00 | 0.00 |
| P4 | LHD | 4.39 | 0.00 | 0.00 | 0.00 | 0.00 | 0.00 | 0.00 | 1.10 |
| P5 | LHD | 0.00 | 0.00 | 63.10 | 100.00 | 0.00 | 0.00 | 0.00 | 12.67 |
| P6 | RHD | 0.00 | 0.00 | 0.00 | 0.00 | 0.00 | 0.00 | 0.00 | 0.00 |
| P7 | LHD | 0.00 | 0.00 | 0.00 | 0.00 | 0.00 | 0.00 | 0.00 | 0.00 |
| P8 | RHD | 0.00 | 0.00 | 0.00 | 0.00 | 0.00 | 69.82 | 15.37 | 7.67 |
| P9 | LHD | 0.00 | 0.00 | 0.00 | 0.00 | 0.00 | 0.00 | 0.00 | 0.00 |
| P10 | LHD | 0.04 | 0.00 | 0.00 | 0.00 | 0.00 | 0.00 | 0.00 | 0.01 |
| P11 | RHD | 0.00 | 0.00 | 0.00 | 0.00 | 0.00 | 0.00 | 0.00 | 0.00 |
| P12 | RHD | 0.00 | 0.00 | 0.00 | 0.00 | 4.28 | 100.00 | 92.17 | 16.32 |
| P13 | LHD | 0.00 | 0.00 | 0.67 | 0.00 | 0.00 | 0.00 | 0.00 | 0.07 |
| P14 | LHD | 0.00 | 0.00 | 0.00 | 71.98 | 0.00 | 0.00 | 0.00 | 4.29 |
| P15 | LHD | 0.00 | 0.00 | 0.00 | 0.00 | 0.00 | 0.00 | 0.00 | 0.00 |
| P16 | RHD | 0.00 | 0.00 | 0.00 | 0.00 | 2.89 | 87.82 | 0.00 | 8.77 |
| P17 | LHD | 0.00 | 0.00 | 0.00 | 0.00 | 0.00 | 0.00 | 0.00 | 0.00 |
| P18 | RHD | 0.00 | 0.00 | 0.00 | 0.00 | 0.00 | 0.00 | 0.00 | 0.00 |
| P19 | LHD | 0.00 | 0.00 | 0.00 | 0.00 | 0.00 | 0.00 | 0.00 | 0.00 |
| P20 | LHD | 0.00 | 0.00 | 0.00 | 0.00 | 0.00 | 0.00 | 0.00 | 0.00 |
| P21 | LHD | 0.00 | 0.00 | 0.00 | 0.00 | 0.00 | 0.00 | 0.00 | 0.00 |
| P22 | LHD | 0.00 | 0.00 | 28.06 | 7.13 | 0.00 | 0.00 | 0.00 | 3.41 |
| P23 | LHD | 0.00 | 0.00 | 9.84 | 51.74 | 0.00 | 0.00 | 0.00 | 4.13 |
| P24 | RHD | 0.00 | 0.00 | 0.00 | 0.00 | 0.00 | 0.00 | 0.00 | 0.00 |
| P25 | RHD | 0.00 | 0.00 | 0.00 | 0.00 | 0.00 | 0.00 | 0.00 | 0.00 |
| P26 | RHD | 0.00 | 0.00 | 0.00 | 0.00 | 0.00 | 0.00 | 0.00 | 0.00 |
| P27 | LHD | 0.00 | 0.00 | 0.00 | 0.00 | 0.00 | 0.00 | 0.00 | 0.00 |
| P28 | LHD | 0.00 | 0.00 | 0.00 | 0.00 | 0.00 | 0.00 | 0.00 | 0.00 |
| P29 | LHD | 0.00 | 0.00 | 0.00 | 0.00 | 0.00 | 0.00 | 0.00 | 0.00 |
| P30 | RHD | 0.00 | 0.00 | 0.00 | 0.00 | 0.00 | 0.00 | 0.00 | 0.00 |
| P31 | LHD | 0.00 | 0.00 | 12.71 | 100.00 | 0.00 | 0.00 | 0.00 | 7.31 |
| P32 | RHD | 0.00 | 0.00 | 0.00 | 0.00 | 0.00 | 0.00 | 0.00 | 0.00 |
| P33 | RHD | 0.00 | 0.00 | 0.00 | 0.00 | 0.00 | 0.00 | 0.00 | 0.00 |
| P34 | LHD | 0.00 | 0.00 | 0.00 | 0.00 | 0.00 | 0.00 | 0.00 | 0.00 |
| P35 | RHD | 0.00 | 0.00 | 0.00 | 0.00 | 0.00 | 0.00 | 0.00 | 0.00 |
| P36 | LHD | 0.00 | 0.00 | 0.00 | 0.00 | 0.00 | 0.00 | 0.00 | 0.00 |
| P37 | RHD | 0.00 | 0.00 | 0.00 | 0.00 | 0.00 | 0.00 | 0.00 | 0.00 |
| P38 | LHD | 0.00 | 0.00 | 0.00 | 0.00 | 0.00 | 0.00 | 0.00 | 0.00 |
| P39 | LHD | 8.19 | 0.00 | 0.00 | 0.00 | 0.00 | 0.00 | 0.00 | 2.05 |
| P40 | LHD | 0.00 | 0.00 | 0.00 | 0.00 | 0.00 | 0.00 | 0.00 | 0.00 |
| P41 | RHD | 0.00 | 0.00 | 0.00 | 0.00 | 0.00 | 0.89 | 78.44 | 5.35 |
| P42 | LHD | 0.00 | 0.00 | 0.00 | 0.00 | 0.00 | 0.00 | 0.00 | 0.00 |
| P43 | RHD | 0.00 | 0.00 | 0.00 | 0.00 | 0.00 | 0.00 | 20.49 | 1.37 |
| P44 | RHD | 0.00 | 0.00 | 0.00 | 0.00 | 0.00 | 0.00 | 0.00 | 0.00 |
| P45 | LHD | 0.00 | 0.00 | 0.00 | 0.00 | 0.00 | 0.00 | 0.00 | 0.00 |

Notes: LHD = left hemisphere damage, RHD = right hemisphere damage, L = left, R = right, ACC = anterior cingulate cortex, a = anterior, SMG = supramarginal gyrus, RPFC = rostral prefrontal cortex

Supplemental Table 8. Differences between Groups in Language Network Connectivity

| **Without controlling for lesion volume** | | | |
| --- | --- | --- | --- |
| Analysis Unit | Test Statistic | *p* value | *q* value (FDR correction) |
| Cluster 1 (of 21) | *F*_2,42_ = 5.77 | 0.006 | 0.129 |
| LIFGop-LSTG | *t*_43_ = 4.27 | < 0.001 | 0.003 |
| LIFGop-LpSTG | *t*_43_ = 3.99 | < 0.001 | 0.003 |
| LIFGop-LSTpole | *t*_43_ = 3.32 | 0.002 | 0.016 |
| LIFGtri-LpSTG | *t*_43_ = 3.60 | < 0.001 | 0.022 |
| LIFGorb-LpSTG | *t*_43_ = 2.80 | 0.008 | 0.103 |
| LIFGop-RSTG | *t*_43_ = 2.15 | 0.037 | 0.111 |
| LIFGorb-LSTpole | *t*_43_ = 2.50 | 0.016 | 0.148 |
| LIFGtri-LSTG | *t*_43_ = 2.24 | 0.031 | 0.166 |
| LIFGtri-LSTpole | *t*_43_ = 2.02 | 0.049 | 0.166 |
| Cluster 2 (of 21) | *F*_2,42_ = 4.23 | 0.021 | 0.222 |
| *n.s.* | | | |
| **Controlling for lesion volume** | | | |
| Cluster 1 (of 21) | *F*_2,41_ = 5.59 | 0.007 | 0.150 |
| LIFGop-LSTG | *t*_42_ = 4.25 | < 0.001 | 0.003 |
| LIFGop-LpSTG | *t*_42_ = 3.98 | < 0.001 | 0.004 |
| LIFGop-LSTpole | *t*_42_ = 3.28 | 0.002 | 0.019 |
| LIFGtri-LpSTG | *t*_42_ = 3.58 | < 0.001 | 0.024 |
| LIFGorb-LpSTG | *t*_42_ = 2.77 | 0.008 | 0.112 |
| LIFGop-RSTG | *t*_42_ = 2.13 | 0.039 | 0.118 |
| LIFGorb-LSTpole | *t*_42_ = 2.45 | 0.018 | 0.166 |
| LIFGtri-LSTG | *t*_42_ = 2.25 | 0.029 | 0.179 |
| Cluster 2 (of 21) | *F*_2,41_ = 4.18 | 0.022 | 0.235 |
| *n.s.* | | | |

Notes: Positive *t* values indicate connections that are significantly stronger in individuals with right hemisphere compared to left hemisphere damage. *n.s.* denotes no significant connections before (or after) FDR correction. IFGop = inferior frontal gyrus, pars opercularis; IFGorb = IFG, pars orbitalis; IFGtri = IFG, pars triangularis; L = left; p = posterior; R = right; STG = superior temporal gyrus; STpole = superior temporal pole.


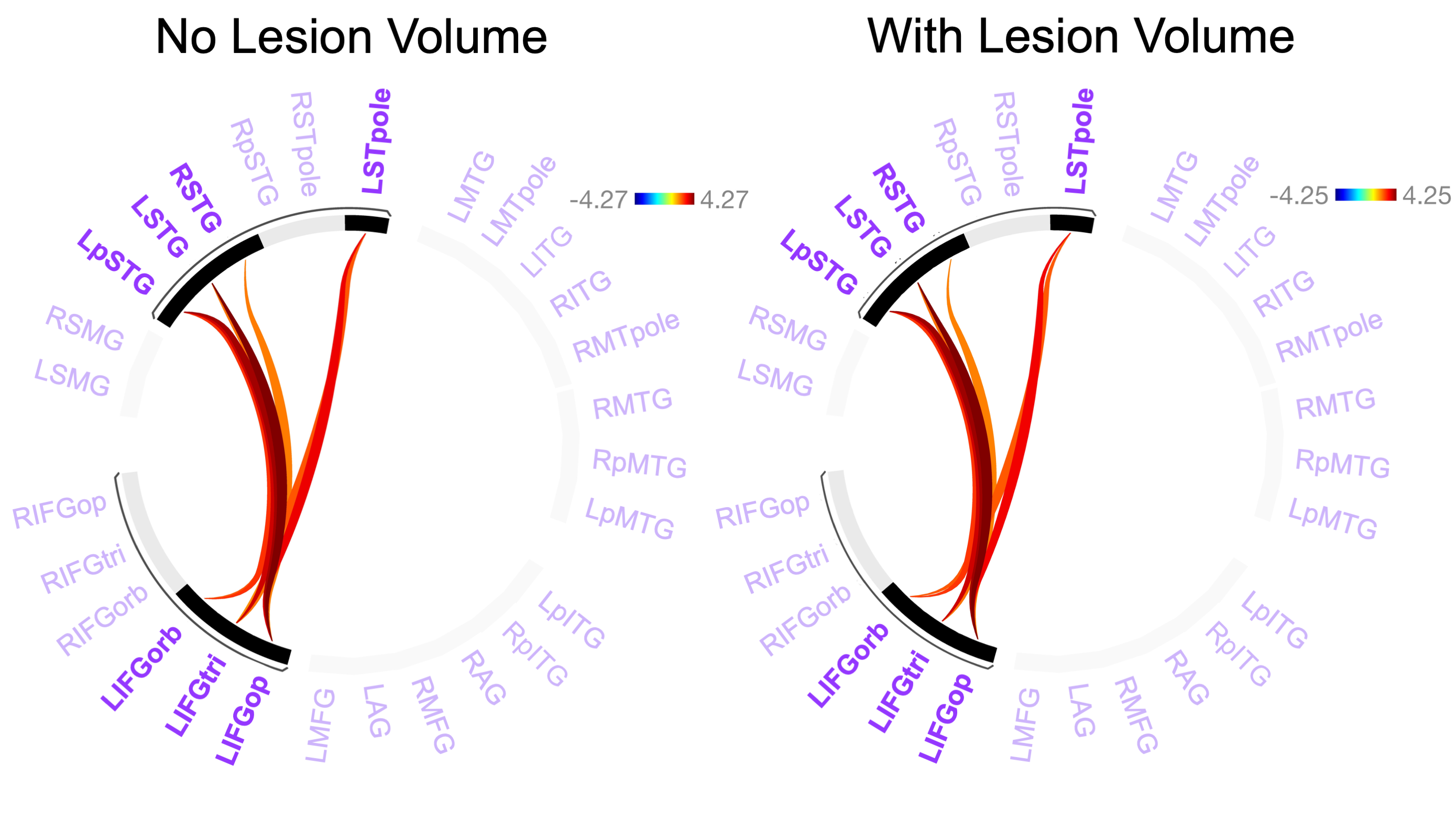


Supplemental Figure 1. Significant differences between groups in language network connectivity at a cluster-level threshold of *p* < 0.001. Warmer colors indicate connections that are significantly stronger in individuals with right hemisphere compared to left hemisphere damage. Region names included in significant connections are in bold font. Language network regions are in purple font. Region labels: AG = angular gyrus; IFGop = inferior frontal gyrus, pars opercularis; IFGorb = IFG, pars orbitalis; IFGtri = IFG, pars triangularis; ITG = inferior temporal gyrus; L = left; MFG = middle frontal gyrus; MTG = middle temporal gyrus; MTpole = middle temporal pole; p = posterior; R = right; SMG = supramarginal gyrus; STG = superior temporal gyrus; STpole = superior temporal pole.
